# Correlation between climatic environment and characteristic components of 14 kinds of huajiao by thermal analysis techniques, GC‐MS and HS‐IMS

**DOI:** 10.1002/fsn3.4126

**Published:** 2024-05-03

**Authors:** Dandan Chang, Yu Yang, Feiyan Tao, Yu Ding, Meiling Jian, Qinwan Huang

**Affiliations:** ^1^ Research and Development Centre China Tobacco Sichuan Industrial Co., Ltd. Chengdu China; ^2^ Sichuan Sanlian New Material Co., Ltd. Chengdu China; ^3^ School of Pharmacy Chengdu University of Traditional Chinese Medicine Chengdu China

**Keywords:** climatic environment, GC‐MS, HS‐IMS, red huajiao(Sichuan pepper), thermal analysis, VOC composition analysis

## Abstract

Huajiao (*Zanthoxylum bungeanum* Maxim.) is extensively cultivated in various countries, including China, Korea, and India, owing to its adaptability to diverse environments. This study comprehensively analyzed the volatile substance composition of 14 varieties of red huajiao with distinct geographical origins. Thermal analysis methods, gas chromatography‐mass spectrometry (GC‐MS), and headspace‐ion mobility chromatography (HS‐IMS) were employed to evaluate the total volatile substance composition and content. The study revealed minor variations in water content, volatile matter content, and fractions among the geographically sourced huajiao samples. Utilizing correlation analysis based on GC‐MS and orthogonal partial least squares discriminant analysis (OPLS‐DA) with HS‐IMS, a robust classification method for the 14 types of huajiao was developed. Applying the variable importance in the projection (VIP) method, seven distinctive components were identified as potential markers for distinguishing the geographical origins of red huajiao. By integrating climatic and topographical factors of the 14 huajiao varieties, the correlation analysis of GC‐MS, and OPLS‐DA classification outcomes from HS‐IMS elucidated the influence of geo‐environmental factors on huajiao components and contents. This research provides insights into the impact of diverse geographic environments on the constituents and characteristics of huajiao. It offers valuable guidance for selecting optimal cultivation locations to enhance huajiao quality, aiding consumers in making informed choices.

## Graphical abstract

In this study, the volatile compositions of 14 red huajiao varieties from different origins were comprehensively analyzed. A robust classification of the 14 huajiao species was performed using GC‐MS‐based correlation analysis and HS‐IMS‐based (OPLS‐DA). The correlation analysis of GC‐MS and HS‐IMS‐based OPLS‐DA classification results elucidated the influence of geo‐environmental factors on the composition and content of the huajiao.

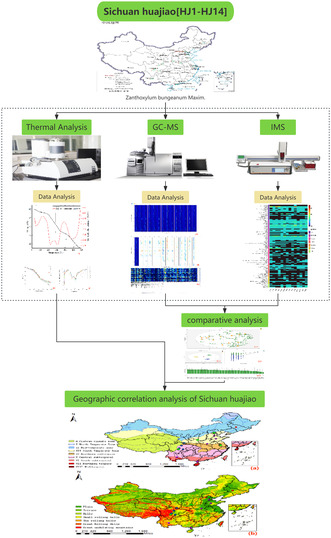

## 1 INTRODUCTION

Huajiao (*Zanthoxylum bungeanum* Maxim.), commonly known as “red huajiao” due to its vibrant red color, holds a significant place in both traditional Chinese medicine and culinary tradition (Bao et al., 2023). With nearly a millennium of culinary history in China, references to huajiao can be traced back to ancient texts such as “Erya,” which is referred to as “Hui” and “Dajiao.” The renowned “Shennong Ben Cao Jing” categorizes it as “Qin huajiao” of middle grade and “Shu huajiao” of lower quality, characterizing its flavor as “pungent” and “warm”. Owing to its distinct volatile organic compounds, huajiao embodies the quintessential “numbness” in Chinese cuisine, especially in the iconic Sichuan dishes. The escalating market demand and economic potential of red huajiao have spurred widespread cultivation in China and other East Asian nations (Zhuo et al., 2020). Revered as the essence of Sichuan cuisine, the “numbness” of huajiao emanates from its volatile organic compounds, pivotal characteristics, and index components (Ma et al., 2019). These volatile organic compounds (VOCs) are intrinsically tied to their geographical location, rendering them notably diverse and complex. Consequently, distinct geographical regions yield distinctive and emblematic huajiao varieties (X. Yang, 2008). For instance, the “Hancheng Dahongpao” boasts high quality due to its origins in a southern temperate zone characterized by concurrent rain and heat and pronounced rainfall seasonality. Conversely, the famed “Hanyuan red huajiao” hails from a subtropical area, basking in ample sunshine (Sun et al., 2020). The resultant variability in the climatic milieu translates into diverse market reputations for different huajiao varieties. Previously, the volatile characteristic components of various geographical indications of huajiao have been analyzed in related works of literature (Feng et al., 2022). However, there is still research value in exploring the correlation between the characteristic components of different huajiao and the geographical environment.

This study meticulously selected and analyzed 14 distinct huajiao varieties, each sporting a unique geographical indication and holding a national production lot number. Geographical indications encapsulate the designated area of origin, a confluence of natural and human factors that confer unique attributes and advantages upon the corresponding product (Brinckmann, 2013). As the defining components and quality markers of huajiao, the content and composition of VOCs stand intimately intertwined with the geographical milieu (Epping & Koch, 2023). In the realm of VOC analysis, an array of techniques, including GC‐MS, selective gas sensors, electronic nose, spectroscopy, miniaturized gas chromatography, ion mobility spectrometry (IMS), and direct injection mass spectrometry (DIMS), find application in huajiao analysis (Obi Johnson et al., 2019). GC‐MS, an established technique, has been upheld as the gold standard for volatile organic compound detection (Chambers et al., 2011). On the other hand, ion mobility spectrometry, an emerging method, offers the advantage of direct global IMS profile comparison, enhancing efficiency in screening and characterizing VOCs compared to GC‐MS (Xing et al., 2022). Meanwhile, thermal analysis techniques, often applied in material science, illuminate the thermal stability of components, a facet pertinent to VOC content (Fernández et al., 2012).

In this study, we used thermal analysis combined with GC‐MS and HS‐IMS to determine the components and related contents of the volatile organic compound components of 14 kinds of huajiao, and thermogravimetric analysis was applied to analyze the total volatile organic compound contents and thermal stability of the huajiao. The GC‐MS data were analyzed for qualitative identification and correlation analysis, and the HS‐IMS data were analyzed using OPLA‐DA analysis and VIP analysis to find the relevant quality markers of the 14 kinds of huajiao. Through the above analysis, we searched for the environmental factors of the geographical indications of huajiao, highlighted the aroma substances and varietal advantages of huajiao, and explored the dependence of the volatile organic compounds of huajiao on the geographical environment.

## 2 MATERIALS AND METHODS

### 2.1 Samples and reagents

**FIGURE 1:**
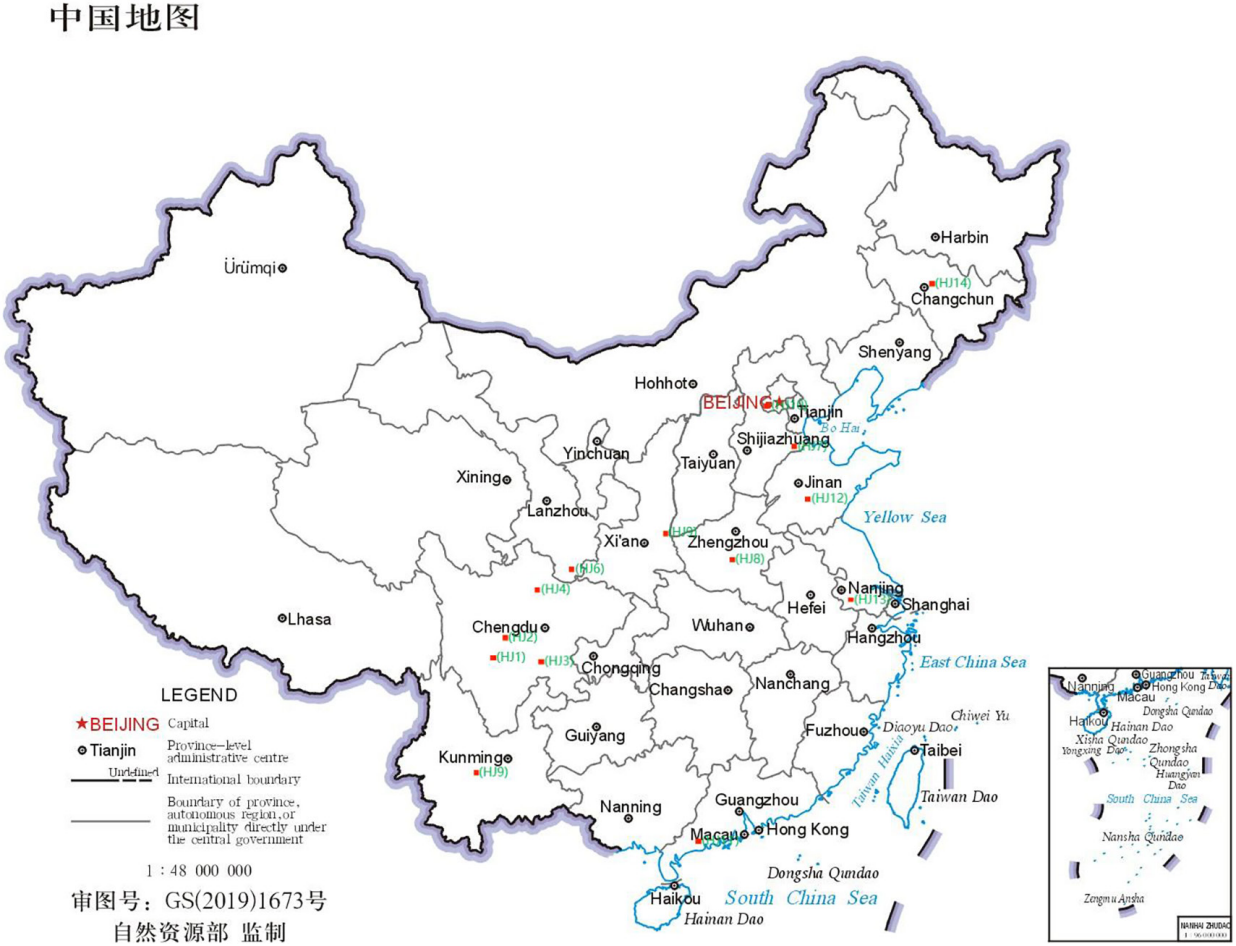
Map of sample collection sites HJ1–HJ14.

A total of 14 distinct varieties of “red huajiao” with diverse geographical indications were meticulously collected from various regions across China. These included Ya'an (Tianquan County) huajiao (HJ1), Hanyuan huajiao (HJ2), Zigong huajiao (HJ3), Maowen huajiao (HJ4), Hancheng huajiao (HJ5), Wudu huajiao (HJ6), Cangzhou huajiao (HJ7), Zhumadian huajiao (HJ8), Kunming huajiao (HJ9), Shunyi huajiao (HJ10), Maoming huajiao (HJ11), Dezhou huajiao (HJ12), Taizhou huajiao (HJ13), and Changchun huajiao (HJ14). Details of the 14 huajiao species are given in Table 1, and the approximate range of coordinates is shown in Figure 1. To facilitate analysis, all huajiao samples were meticulously ground into powder using a micro‐mill and subsequently passed through a 40‐mesh sieve, and the huajiao samples used in the following experiments were all powdered samples.

**TABLE 1:** HJ1–HJ14 origin and batch number.

Number	Origin	Longitudes	Dimension (math.)	Product standard number	Product manufacturing license number
HJ1	Sichuan‐Ya'an	102°40′42″ E	29°21′6″ N	GB/T 30391	—
HJ2	Sichuan‐Hanyuan	103°0′0″ E	29°58′48″ N	Q/CYJ 0011S	
HJ3	Sichuan‐Zigong	104°27′36″ E	29°13′48″ N	Q/BWZ 0001S	SC10251031100015
HJ4	Sichuan‐Maoxian	103°51′13″ E	31°40′55″ N	GB/T 30391	—
HJ5	Shaanxi‐Hancheng	110°16′32″ E	35°28′45″ N	Q/HDM 0020S	—
HJ6	Gansu‐Wudu	104°55′35″ E	33°23′32″ N	GB/T 30391	SC10362262100645
HJ7	Hebei‐Cangzhou	116°49′48″ E	38°19′48″ N	Q/HHSD 0013S	SC10113090400012
HJ8	Henan‐Zhumadian	114°1′12″ E	32°58′48″ N	Q/ZWST 0005S	SC10341170200031
HJ9	Yunnan‐Kunming	102°6′0″ E	24°13′48″ N	GB/T 15691	SC10353011108126
HJ10	Beijing‐Shunyi	116°39′0″ E	40°7′48″ N	Q/SYBWT 0001	—
HJ11	Guangdong‐Maoming	110°11′24″ E	21°13′12″ N	Q/WFY 0001S	SC10344090400335
HJ12	Shandong‐Dezhou	116°39′0″ E	40°7′48″ N	GB/T 15691	SC10337140100260
HJ13	Jiangsu‐Taizhou	119°22′48″ E	32°0′36″ N	Q/JSWM 0001S	SC10132128101591
HJ14	Jilin‐Changchun	125°21′0″ E	43°52′48″ N	GB/T 15691	SC12122010310505

*Note*: The last two columns of the table are the licenses related to the production and sale of food in China.

A C_7_–C_40_(1000 mg/mL) n‐alkane standard mixture for GC‐MS analysis was procured from ANPEL‐TRACE Standard Technical Services CO. (Shanghai, China). A n‐hexane, employed for GC‐MS analysis, was obtained from Chengdu Cologne Chemical Co. (Chengdu, China). The thermogravimetric analysis was performed using a NETZSCH STA 449 F3 Jupiter® thermal analyzer (Germany). For qualitative identification of volatile components, the GC‐MS setup consisted of an Agilent 8890 GC coupled with an Agilent 59778B GC‐MSD. The HS‐IMS apparatus utilized for the analysis was the FlavourSpec®, G.A.S. (Dortmund, Germany), equipped with a syringe and an automated headspace research sampler unit.

### 2.2 Thermogravimetric analysis

Thermogravimetric analysis was employed to examine the thermal behavior of HJ1–HJ14 samples. While thermal analysis is not commonly used for compositional analysis of volatile organic compounds, it can provide insights into the moisture and volatile content percentage of substances, offering potential value for certain applications (Bravo et al., 2020). Thermogravimetric analysis was carried out on HJ1–HJ14, and the samples were sieved through 150 mesh sieves. In a typical procedure, 5 mg of sample was taken for thermogravimetric analysis. The initial temperature was 25°C, after which the temperature was ramped up to 10°C every minute until it reached 600°C. The sample reference was an empty aluminum crucible, the air ambiance was nitrogen, and each sample was duplicated three times. Raw data were processed using system programs and Excel, and processed data were visualized and analyzed using Origin 2019b.

### 2.3 GC‐MS

GC‐MS was employed for the qualitative analysis of HJ1–HJ14 samples, and the relevant conditions were improved based on the results of existing experiments (W. Zhang et al., 2019). In a typical procedure, 10 g of huajiao sample was accurately weighed into 30 mL of n‐hexane, extracted by ultrasonic machine (50°C, 230 W) for 120 min, vortexed for 10 min after 8 h of rest, and the supernatant was taken through 0.22 μL microporous filter membrane as a sample for GC‐MS. The capillary column was used DB‐5 (60 m × 0.25 mm i.d. ×0.25 μm d.f.). The inlet temperature was 250°C, and the sample volume was 1 μL per injection with an injection split ratio of 15:1. The carrier gas was helium‐fed at a constant flow of 1 mL/min. The temperature increase conditions of the program were as follows: hold 60°C for 2 min, increase to 280°C at a rate of 8°C/min, and hold for 25 min. The ion source temperature was 230°C and operated in electron impact (EI) mode at 70 eV. The solvent delay time was 4.8 min. A full scan mode was used in the range of 33–400 *m/z*.

In this study, we used the retention index (RI) calculation method to qualitatively analyze the unknown volatile organic compounds of HJ1–HJ14 by comparing the retention indices of the analytical results with the retention indices of the relevant literature and the data of NIST14 software (Wei et al., 2014). In this study, the qualitative VOCs were not quantitatively analyzed, so we did not calculate the absolute content of various VOCs in huajiao. The relative content ratios of volatile organic compounds were determined from the results of the qualitative analysis. The relative content quantification was performed by comparing the area of a single peak, applying the 1% heptane peak as a control peak to convert the peak area ratio of each qualitative height in the 14 samples, which was used as a supplemental analysis because of the imprecision of the calculation method.

### 2.4 HS‐IMS

Headspace‐ion mobility chromatography (HS‐IMS) was employed to analyze VOCs in HJ1–HJ14 samples. In the headspace injection program, 100 mg of the huajiao were weighed accurately into a 20 mL headspace vial and incubated at 60°C for 10 min. The headspace injection volume was 250 μL, and the injection port temperature was 60°C. The volatile organic compounds of the huajiao were separated on a MXT‐WAX capillary column (0.28 mm × 0.25 μm × 15 m) and transported to an IMS detector at a column temperature of 60°C. The carrier gas was high‐purity nitrogen with a purity greater than 99.999%, and the carrier gas flow rate was initially 2 mL/min for 2 min and was increased to 10 mL/min for 8 min. At the 10th min, the carrier gas flow rate was increased to 100 mL/min and continued until the 40‐min mark. The separated components were ionized in the ionization chamber of IMS. The ionized details were transferred to a drift tube at 45°C using 150 mL/min of high‐purity nitrogen and detected in a detector at 45°C.

The results of the IMS data were analyzed using relevant software. Laboratory Analytical Viewer (LAV) was used to view the ion mobility spectra; NIST and IMS databases were used for the qualitative analysis of the substances; the Gallery Plot plugin was used to draw the fingerprint spectrum of the samples to visually compare the differences of volatile organic compounds between different models; and the Reporter plugin could directly The Reporter plugin can directly compare the differences in the fingerprint spectrum between samples. We performed quantitative analysis by comparing the retention times of IMS libraries (GAS) and experimental results (Kirk et al., 2019).

### 2.5 Data analysis

Thermogravimetric maps were plotted using Origin 2019b (Origin Lab Corporation, Northampton, MA, USA) software. The fingerprints of IMS were plotted using the HS‐IMS system software to compare the differences in the VOCs content of HJ1–HJ14. The software also plotted two‐dimensional plots of the huajiao, with each point on the field representing a peak. These plots were characterized by retention time, relative drift time, and signal intensity (Capitain & Weller, 2021). Climatic and topographic maps of HJ1–HJ14 were prepared using Arc GIS software, and all geographic information data were obtained from the Center for Resource Environmental Science and Data (www.resdc.cn) (Koo et al., 2018). Cluster analysis heat map of GC‐MS and HS‐IMS relative content and histogram of VOCs' percent content were plotted using CHIPLOT software. One‐way ANOVA was performed using SPSS26.0 software, and *p* ≤ .05 was considered significant. Principal component analysis and OPLS‐DA analysis were performed using SIMCA 14.0 software, and correlation analysis plots and permutation test plots were drawn (Shi et al., 2021).

## 3 EXPERIMENTAL RESULTS

### 3.1 Thermogravimetric analysis

**FIGURE 2:**
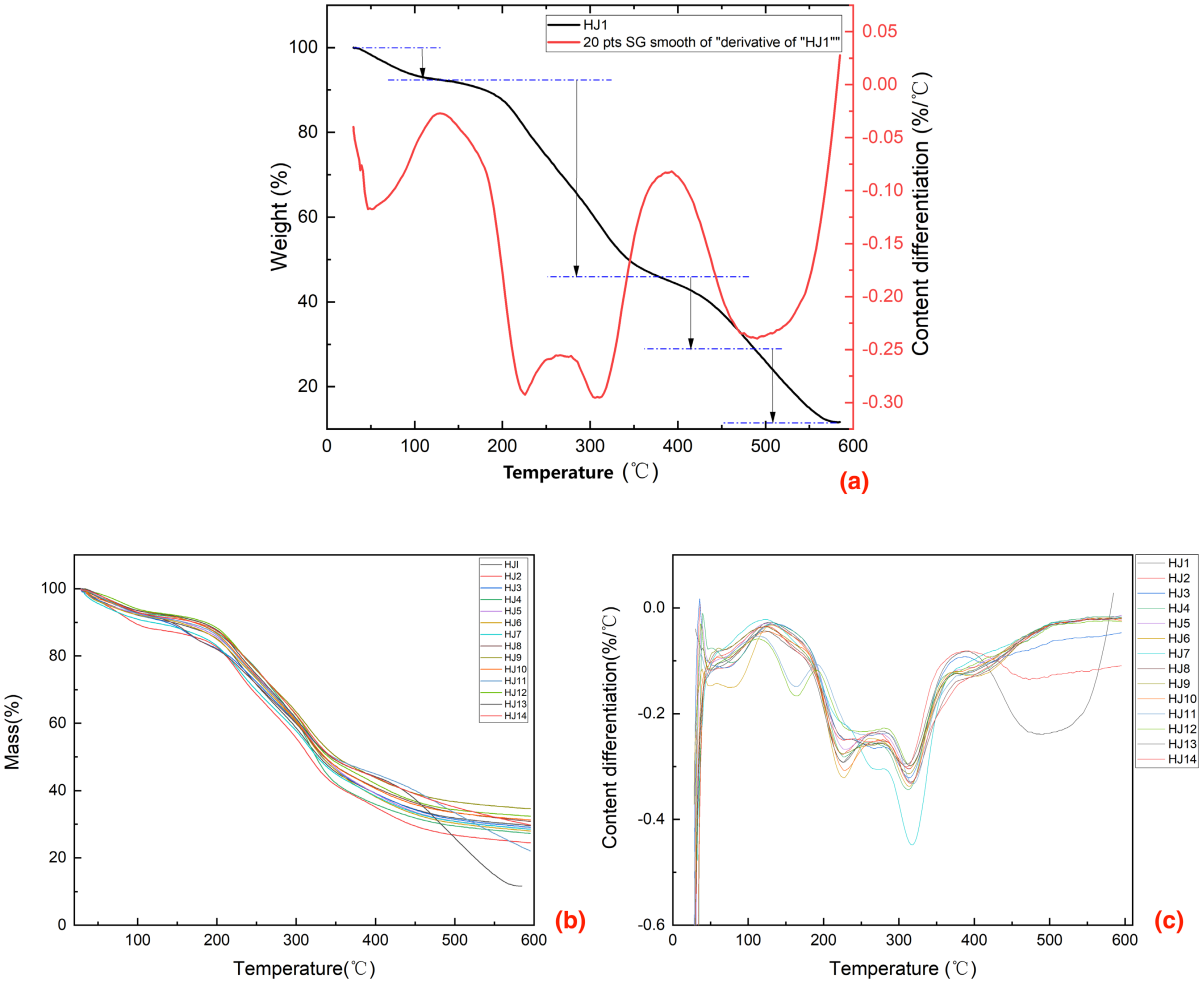
(a) Heat loss curve of HJ1 plotted against 20 times smoothed differential curve. (b) Summary of heat loss diagrams for HJ1–HJ14. (c) Summary of heat loss differential graphs for HJ1–HJ14. (20 smoothing of differential curves using the Savitzky–Golay method)

Thermal analysis technology, as a technique to study the relationship between the structure and properties of substances and the related reaction laws, is now mainly used to study the changes in thermal and other physical parameters that occur when a sense is at a specific temperature (Ma et al., 2017). In the public's choice of huajiao, the degree of aroma intensity has become an essential indicator for judging the quality of huajiao. Aroma intensity is affected by several aspects, including the content and composition of volatile organic compounds. In addition, under different heating temperatures, the degree of aroma dispersion also determines the quality of the spices (Otunola, 2021). To determine the thermal stability of huajiao with different geographical indications, HJ1–HJ14 was analyzed by thermogravimetric analysis. The results are shown in Figure 2. By analyzing the thermogravimetric data of HJ1, it was learned that the heating weight loss phase of the huajiao was divided into four parts (the readings of temperature and weight loss are approximate):

- Stage 1: Water loss (temperature: 20°C–130°C; weight loss: 10%).

In this stage, huajiao samples lost water and other volatile substances.

- Stage 2: Volatile escape (temperature: 130°C–370°C; weight loss: 45%).

During this stage, volatile organic compounds (VOCs) escaped from the samples.

- Stage 3: Pre‐carbonation (temperature: 370°C–470°C; weight loss: 15%).

This stage involved the loss of bound water and decomposition of cellulose.

- Stage 4: Carbonization (temperature: 470°C–600°C; weight loss: 18%).

In the final stage, the structure of huajiao was carbonized and converted to ash.

From Figure 2a, the general trend of HJ1–HJ14 was the same in the first stage and the third stage. In the second stage, the differential curve of weight loss of HJ1–HJ14 has a slight convex peak, and the peak width and peak height are different in huajiao with various geographical indications, which is due to the other contents of terpenes, alcohols, and esters in the volatile VOCs of huajiao. Each volatile organic compound has its thermodynamic properties; the different range of each component makes the thermal stability of huajiao different. This other thermodynamic behavior can be a way to discriminate huajiao (Figure 2b,c). However, the content of volatile organic compounds in huajiao with the same geographical indications may also vary depending on the time of growth, temperature, and precipitation. Therefore, thermogravimetric analysis can only be used as an auxiliary identification method for huajiao with different geographical indications. Sichuan pepper is a common spice widely used in various Chinese dishes. During cooking processes such as boiling, low‐temperature baking, frying, and roasting, VOCs inside the huajiao are released, bringing specific sensory sensations to people (Zhang et al., 2023). The results show that the VOCs of huajiao will be dissipated at 130°C–370°C. Different heating methods of temperature and medium will cause selective excretion of VOCs, resulting in various aromas and feelings. The results of the study showed that the VOCs of huajiao are terpenoids, esters, alcohols, allyl aldehydes, and ketones, of which terpenoids, esters, ketones are lipophilic components (Wu et al., 2023).

Steaming (90°C ~ 110°C) will cause the hydrophilic components to leak out so that the food will emit the compound fruity aroma characteristic of allyl aldehyde components. Low‐temperature roasting (150°C ~ 180°C) will cause the huajiao to lose their moisture and low‐boiling point VOCs, giving the food a more decadent aroma and flavor (Jia et al., 2017). In Chinese cuisine, the frying temperature is 150°C ~ 240°C. Frying huajiao requires a large amount of oil, which leads to water loss and VOC leakage and transfers the aroma of the huajiao to the dishes. The rapid water loss in the huajiao produces a distinctive burnt flavor and makes the dish taste delicious (Ni et al., 2021). Roasting (320°C ~ 370°C) causes huajiao to lose their aroma components quickly and exposes them to the air, adding flavor to food. In addition, Huajiao produces different sensory effects under various cooking methods, bringing people a rich and diverse taste experience.

### 3.2 GC‐MS analysis

**FIGURE 3:**
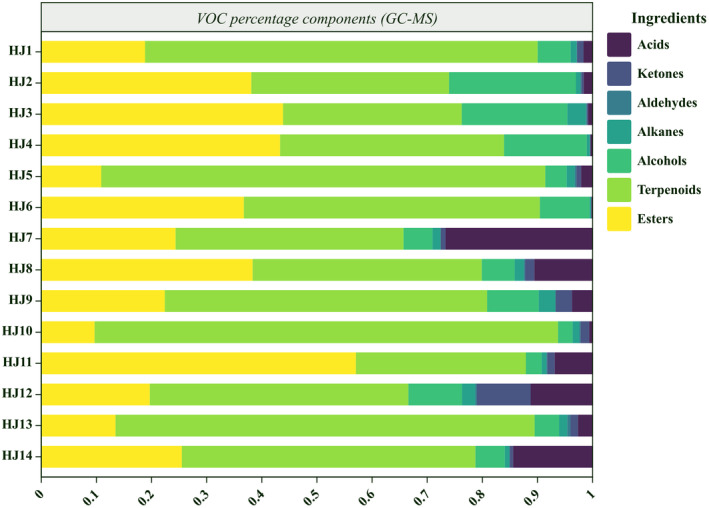
Histogram of components and percentage content of volatile organic compounds HJ1–HJ14 (GC‐MS).

**FIGURE 4:**
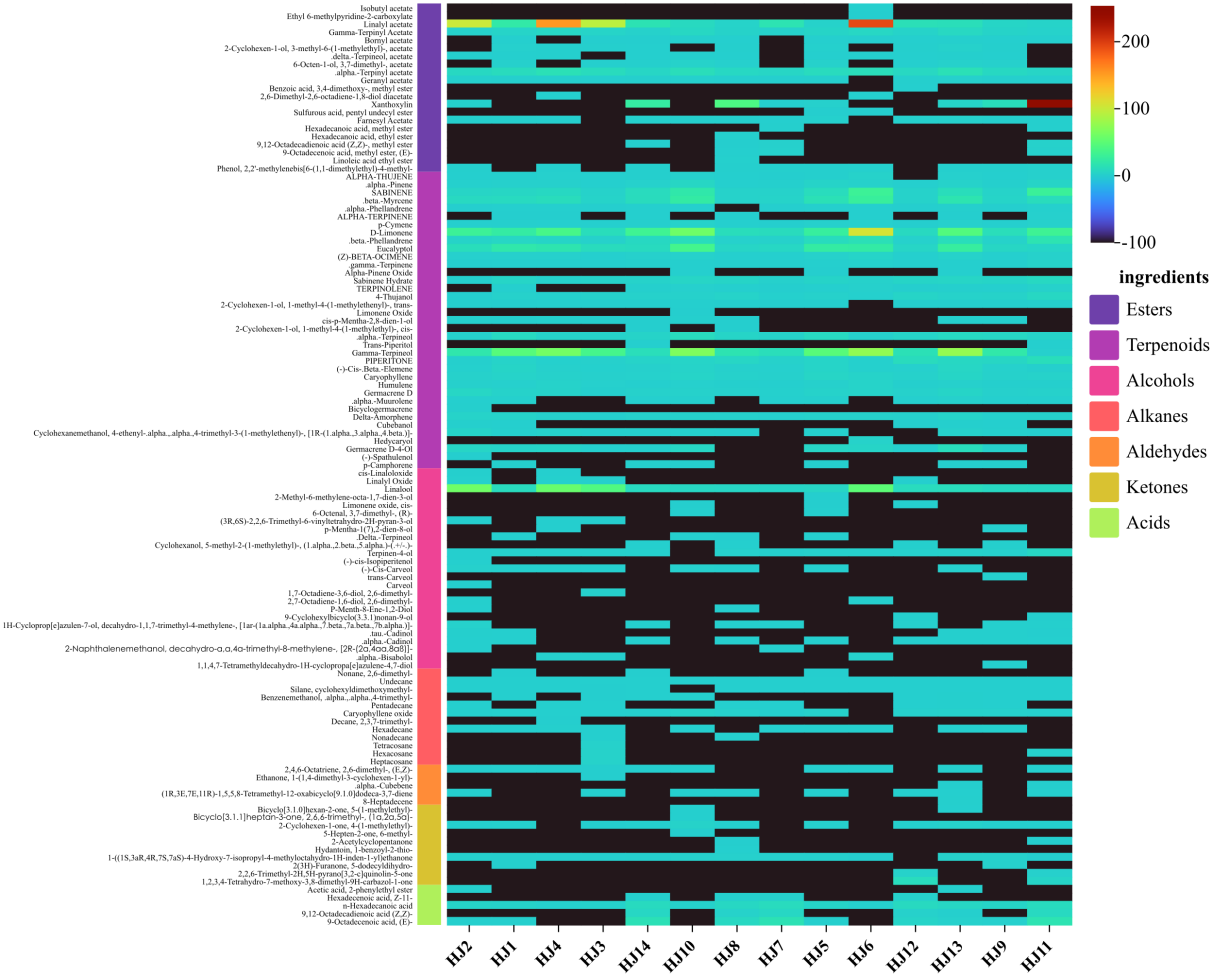
Thermograms (GC‐MS) of volatile organic compounds of HJ1–HJ14. (The relative content of the identification was calculated by comparing the VOCS peak area to that of n‐heptane (10 mg/mL). No “‐100” was detected.)

The qualitative analysis (GC‐MS) of HJ1–HJ14 detected 115 volatiles. These compounds included 21 esters, 37 terpenes, 25 alcohols, 12 alkanes, 5 alkenes, 10 ketones, and 5 acids. For visualization, we summarized the percentage relative content of volatile organic compounds within the HJ1–HJ14 fractions and made a histogram (Figure 3) and a compositional heat map (Figure 4). Among them, terpenes and esters are the main components of VOCs in huajiao, as shown in (Figure 4), the content of terpenes and esters is more than 60% of the total VOCs.The heatmap of the qualitative analysis of HJ1–HJ14 is shown in Figure (4), and the cell with the −100 content represents non‐detection. The relative content of all VOCs was obtained as the ratio of the peak area in the qualitative analysis to the peak area of n‐heptane at 10 mg/L. In HJ1–HJ14, 58, 52, 60, 63, 56, 70, 67, 52, 55, 60, 55, 59, 51, 55 types of volatile organic compounds were detected, respectively. Among them, linalyl acetate and α‐terpinyl acetate were the two most abundant esters; sabinene, β‐myrcene, _D_‐limonene, eucalyptol, and γ‐terpinene were the five terpenoids with the highest percentage of content; linalool was the most abundant alcohol.HJ2, HJ3, HJ4, and HJ6 had a high content expression of linalyl acetate, linalool, and γ‐terpinene, which may be related to the similarity of the geographic environment. Xanthoxylin had very high expression in HJ11, which was related to its location in the tropical monsoon climate zone.

### 3.3 HS‐IMS analysis

Ion mobility chromatography (IMS) serves as a potent separation and diagnostic technique. Acknowledged for its swift responsiveness and cost‐effectiveness, IMS finds prominence in the detection of minute trace compounds across various matrices (Alikord et al., 2022). Divergent from the methodology of GC‐MS, which hinges on chemical morphology for analyte identification, IMS relies on an array of two‐dimensional (2D) topographic profiles (component analysis maps) to shape a comprehensive fingerprint of VOCs during sample detection (Schober et al., 2018). In our study, we harnessed HS‐IMS for the analysis of VOCs across the spectrum of HJ1–HJ14.

**FIGURE 5:**
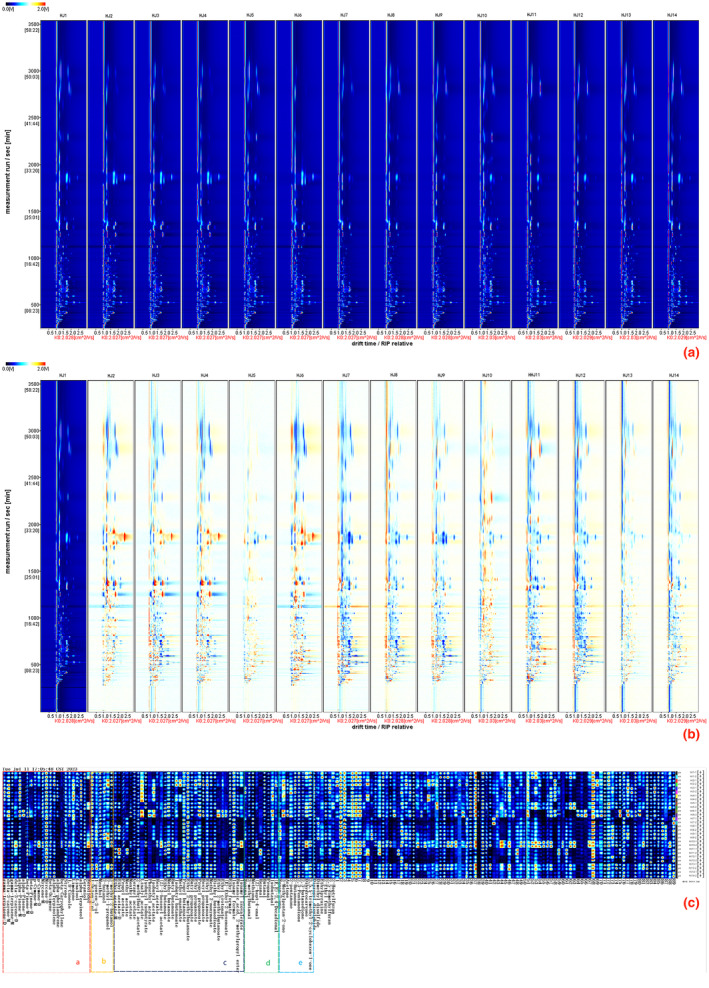
(a) Two‐dimensional spectra of volatile organic compounds of HJ1–HJ14. (b) Differential spectra of volatile organic compounds of HJ1 and HJ2–HJ14. (c) Fingerprints of volatile organic compounds of HJ1–HJ14.

In Figure 5a, a two‐dimensional graphical representation of HJ1–HJ14 unfolds, featuring drift time along the horizontal axis and retention time along the vertical axis. The vertical crimson line on the left symbolizes active ion peaks, while the azure hue constitutes the image's backdrop, accompanied by Ko values ranging between 2.027 and 2.030 cm^2^/Vs. Notably, each point within the graph signifies a distinct VOC, with the intensity of the crimson hue reflecting the relative content of the respective VOC. Figure 5b portrays a two‐dimensional disparity mapping between HJ1 and HJ2–HJ14. Employing parameters like those in Figure 5a, this figure delineates the contrast in VOCs between the latter, HJ2–HJ14, and the former, HJ1. In this visualization, a vivid scarlet hue conveys heightened content, while a blue tone signifies diminished range. The color gradient's depth correlates with the magnitude of the content difference. Notably, the VOCs within “huajiao” were analyzed quantitatively and qualitatively via amalgamating parameters such as Retention Index (RI), retention time, drift time, and Ko value, drawing from the NIST library.

The outcome of our analysis is embodied in Figure 5c, portraying fingerprints of HJ1–HJ14 grounded in our analytical results. Within this construct, a total of 86 VOCs attained preliminary identification, while an additional 89 compounds remain unrecognized. These 86 VOC components can be broadly classified into six categories, namely: 19 terpenes (a), 7 alcohols (b), 31 esters (c), 12 allyl aldehydes (d), 9 ketones (e), and 8 other constituents. For visualization, we summarized the relative percentage and absolute content of each component of HJ1–HJ14 and made histograms of content percentages and cluster analysis heatmaps.

**FIGURE 6:**
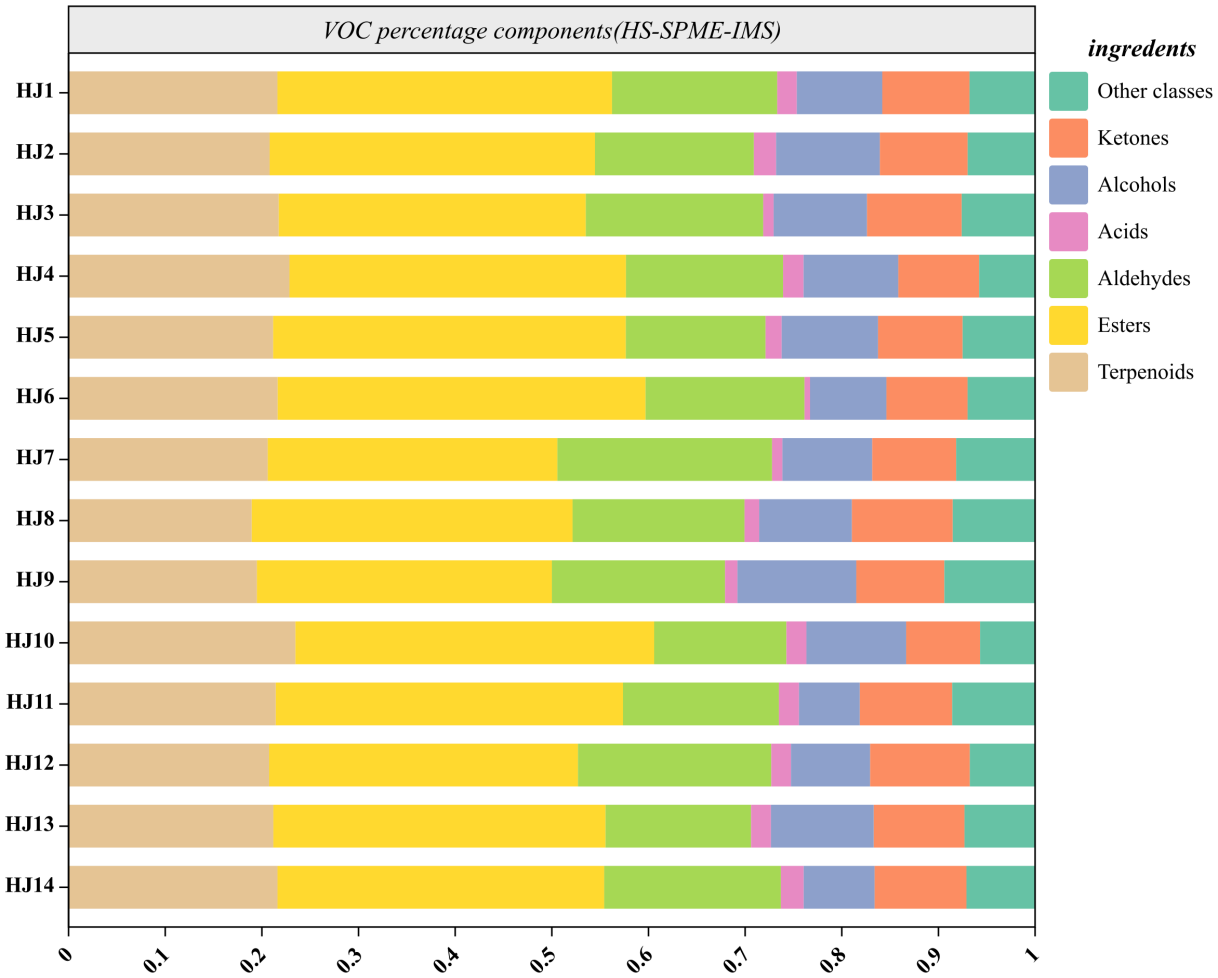
Histogram of percentage content of volatile organic compounds for HJ1–HJ14 (HS‐SPME‐IMS).

**FIGURE 7:**
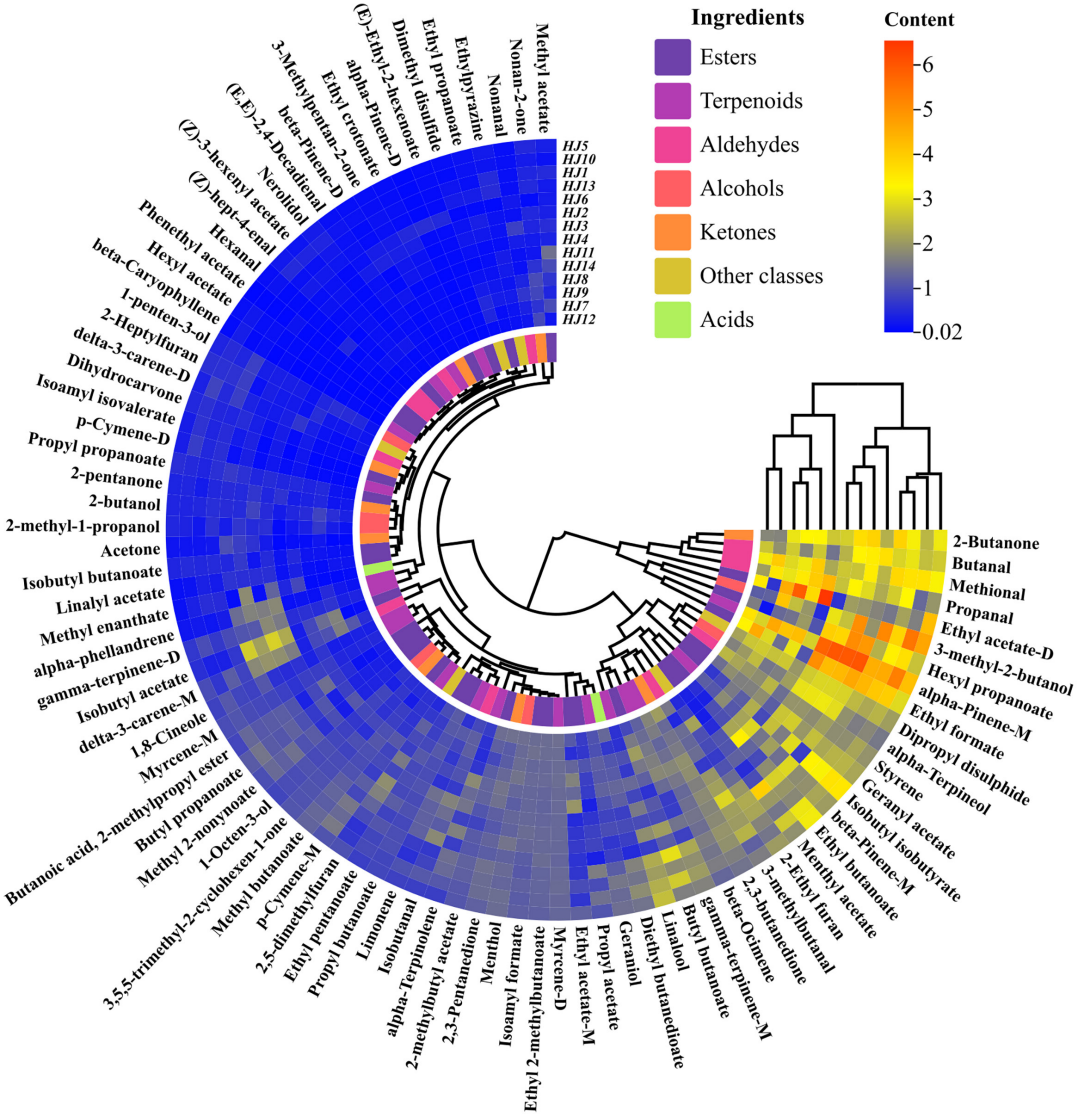
Heat map of cluster analysis of VOC content of HJ1–HJ14 (HS‐SPME‐IMS).

Through Figures 6 and 7, we learned that the VOC content of HJ1–HJ14 was mainly composed of terpenes, esters, alcohols, allyl aldehydes, ketones, and other components. Among them, the terpenes and esters content accounted for more than 50% of the main volatile organic compounds of HJ1–HJ14. Among them, terpenoids include (1,8‐cineole, α‐phellandrene, α‐pinene‐_D_, α‐pinene‐_M_, α‐terpinolene, β‐caryophyllene, β‐ocimene, β‐pinene‐_D_, β‐pinene‐_M_, γ‐terpinene‐_D_, γ‐terpinene‐_M_, geraniol, limonene, linalool, myrcene‐_D_, myrcene‐_M_, nerolidol, p‐cymene‐_D_, p‐cymene‐_M_). Most of the terpenes in HJ7, HJ8, HJ11, and HJ12 were lower than the other huajiao, and HJ6 had a significantly higher content of α‐pinene‐_D_ than the other huajiao. nerolidol, p‐cymene‐_D_ were higher in HJ1, HJ5, HJ10, and HJ13.And α‐terpinolene, limonene in HJ2, HJ3, HJ4, and HJ6, has a high content expression. In addition, the ester content also had a large percentage of total VOCs, including ((E)‐ethyl‐2‐hexenoate, (Z)‐3‐hexenyl acetate, 2‐methylbutyl acetate, butanoic acid, 2‐methylpropyl ester, butyl butanoate, butyl propanoate, ethyl 2‐methylbutanoate, ethyl acetate‐_D_, ethyl butanoate, ethyl crotonate, ethyl formate, ethyl pentanoate, ethyl propanoate, geranyl acetate, hexyl acetate, hexyl propanoate, isoamyl formate, isoamyl isovalerate, isobutyl acetate, isobutyl butanoate, isobutyl isobutyrate, linalyl acetate, menthyl acetate, methyl 2‐nonynoate, methyl acetate, methyl butanoate, phenethyl acetate, propyl acetate, propyl butanoate, propyl propanoate, and ethyl acetate‐_M_). As shown in Figure (6), HJ6 had a significantly higher content of €‐ethyl‐2‐hexenoate, ethyl crotonate, ethyl 2‐methylbutanoate, and phenethyl acetate than other huajiao.HJ2, HJ3, HJ4, and HJ6 had a higher content of isobutyl acetate. The ethyl acetate‐_D_ and ethyl acetate‐_M_ content in HJ8, HJ9, and HJ11 was significantly higher than in other huajiao. The content of hexanal in HJ10 and (Z)‐hept‐4‐enal in HJ5, HJ10, and HJ13 was higher than the other huajiao. Among the ketones, nonan‐2‐one was higher in HJ8, HJ9, and HJ12. HJ2, HJ3, and HJ6 had higher levels of acetone and 3‐methylpentan‐2‐one. In addition, 2‐heptylfuran and ethylpyrazine were detected with higher concentrations in HJ1, HJ5, and HJ13. Dimethyl disulfide was detected with higher levels in HJ7, HJ8, and HJ9.

### 3.4 GC‐MS correlation analysis

**FIGURE 8:**
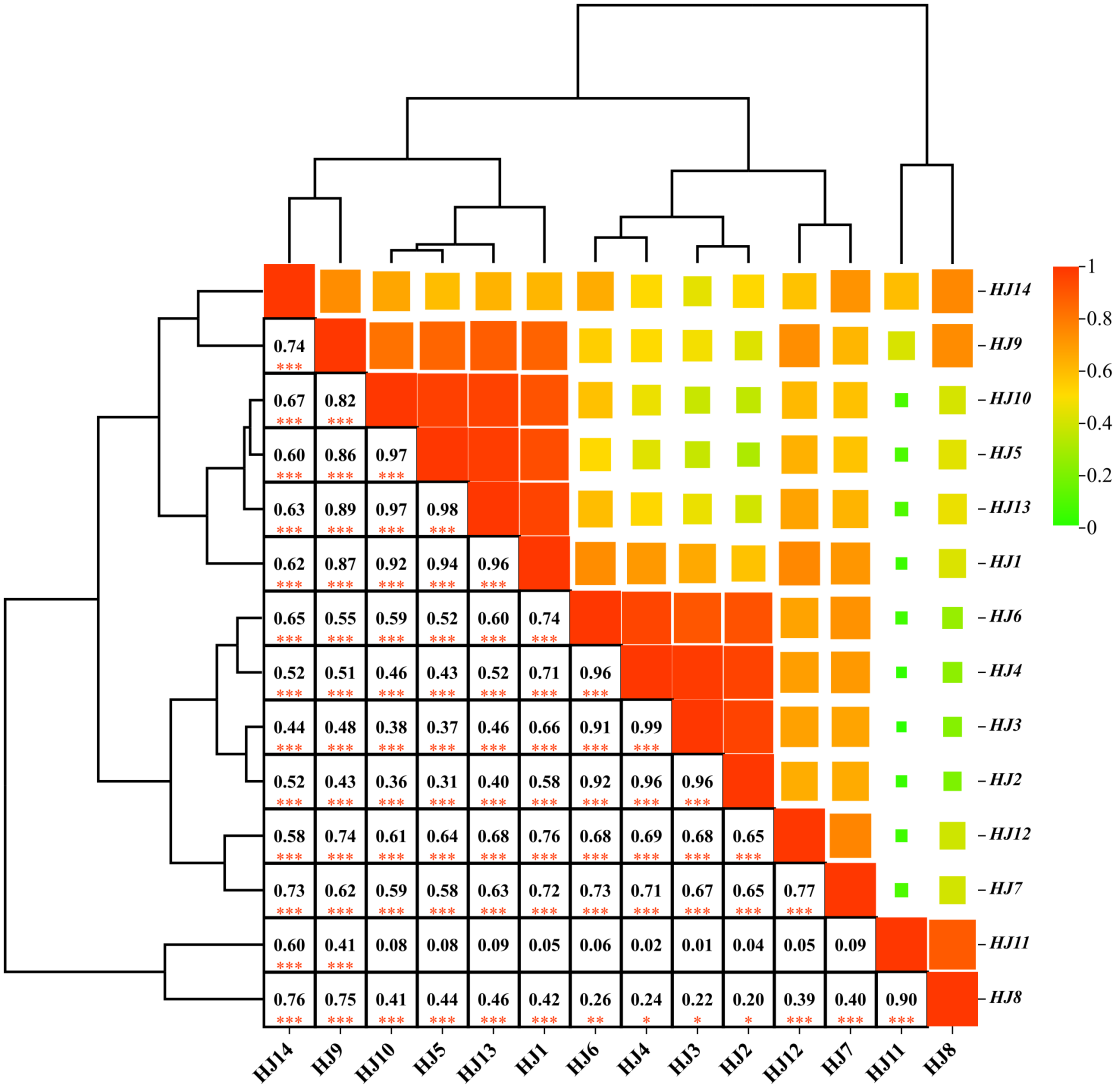
Correlation diagram of cluster analysis of GC‐MS data of HJ1–HJ14. (The more * symbols below the number box in the lower left of the figure, the higher the similarity between the two. * represents the probability that the two are not related *p* ≤ .1, ** represents the probability that the two are not related *p* ≤ .01; *** represents the probability that the two are not related *p* ≤ .001).

Correlation, in its broader sense, entails a measurement of the association between variables. When a change in one variable corresponds with a change in another, this correlation can be positive (in the same direction) or negative (in the opposite direction), indicating a linkage (Armstrong, 2019). Correlation analysis aims to unveil potential associations between two variables and quantify the strength of such relationships (Liljequist et al., 2019). Within our study, correlation analysis was diligently executed on the data spanning HJ1–HJ14. The outcomes are showcased in Figure 8, where the intensity of color directly reflects the potency of the correlation between paired variables. Conventionally, a correlation exceeding .9 signifies a robust correlation, while a correlation surpassing .7 indicates a robust correlation; conversely, correlations below .4 are considered weak (Christmann et al., 2022). The cluster analysis correlation diagram vividly illustrates the interrelation between HJ2, HJ3, HJ4, and HJ6. These varieties, nestled within the subtropical monsoon climate zone and characterized by akin altitudes and topographies, exhibit a striking correlation. Similarly, HJ7 and HJ12 display pronounced correlations, rooted in their habitat within the temperate monsoon climate zone's windward slope, endowed with abundant precipitation and optimal light conditions. Moreover, statistical analysis reveals no significant disparity between HJ1–HJ7, HJ9–HJ10, and HJ12–HJ14. Akinly, HJ8 and HJ11 share no statistically substantial differentiation. These findings suggest that HJ8 and HJ11 likely share a common origin, while the remaining “huajiao” may stem from another source.

### 3.5 HS‐IMS principal components and OPLS‐DA analysis

**FIGURE 9:**
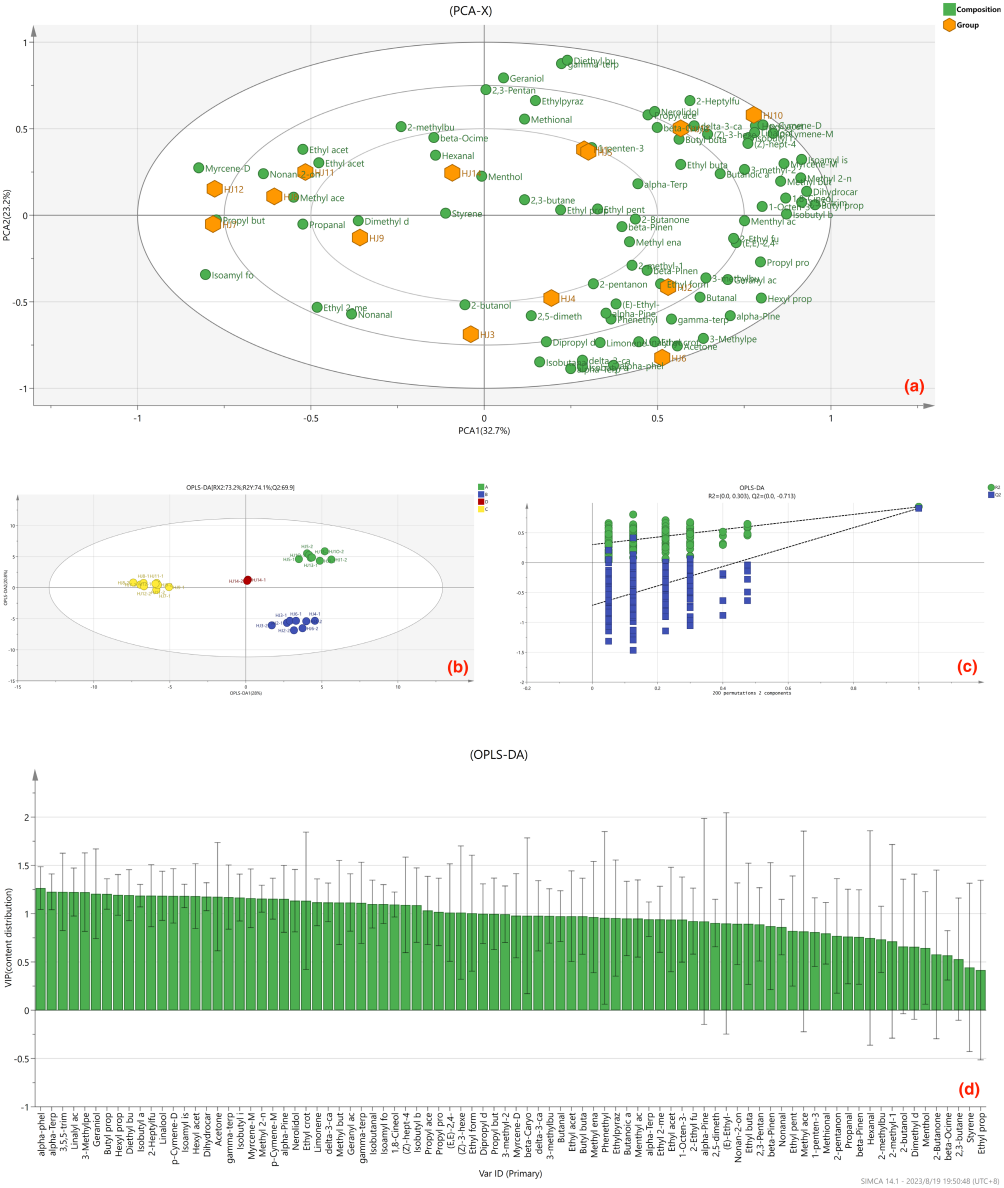
(a) Plot of PCA scores of HJ1–HJ14. (b) OPLS‐DA evaluation score plot for HJ1–HJ14. (c) OPLS‐DA test replacement plot for HJ1–HJ14. (d) OPLS‐DA component VIP score plot for HJ1–HJ14.

HS‐IMS is a technique emerging in recent years for detecting volatile organic compounds. Compared with GC‐MS, HS‐IMS is highly sensitive in detecting volatile organic compounds, has a larger detection width, and can see more volatile organic compounds (Pu et al., 2020). To delineate and differentiate the characteristic aromas pervading HJ1–HJ14, HS‐IMS data underwent Principal Component Analysis (PCA) and Orthogonal Partial Least Squares Discriminant Analysis (OPLS‐DA) treatments. The resultant insights are encapsulated in Figure 8. Figure 9a unveils the PCA‐X score plot of HJ1–HJ14, with principal component 1 accounting for 32.7% and central component 2 accounting for 23.2%. This distribution yields a classification of HJ1–HJ14 into four main categories: HJ2, HJ3, HJ4, and HJ6 cluster in the lower right quadrant; HJ1, HJ5, HJ10, and HJ13 congregate in the upper right quadrant; HJ7, HJ8, HJ9, HJ11, and HJ12 aggregate to the left; HJ14 occupies the center. The composition of volatile organic compounds in HJ1–HJ14 is determined by topographical and environmental factors as well as germplasm factors, and HJ1–HJ14 do not show a high degree of concentration in the PCA scores, which allows each huajiao to have its distinctive aroma components. HJ1 showed a high positive correlation with geraniol, ethylpyrazine, and 2‐heptylfuran, indicating a distinctive rosy, sweet, and cocoa aroma; the other huajiao with their representative aroma components are shown in Table 2. Existing studies have used the multi‐factor quality evaluation method to evaluate the flavor quality of the relevant substances and construct a “flavor profile”, and then analyze the aroma of the substances by comparing the content of the aroma components and evaluating the contribution of the volatile components to the aroma quality, which may become a new method for the study of peppercorns in the study of the characteristic components (Zhao et al., 2023).

**TABLE 2:** Description of the main aromas of HJ1–HJ14.

Kinds	Substance	Fragrance description
	Geraniol Ethylpyrazine 2‐Heptylfuran	Mild, sweet rose scent with a bitter taste.
HJ1	Ethylpyrazine	Nutty, woody, roasted, meaty, fishy, cocoa aromas.
	2‐Heptylfuran	Sweet, dairy aroma with greasy notes of fat.
	α‐Pinene‐_M_	Pine, coniferous, and resinous.
HJ2	Hexyl propanoate	Fresh scent with sweet fruits.
	3‐Methylbutanal	Has a malty aroma.
	Isobutyl acetate	Aroma of raw pear and raspberry.
HJ3	γ‐Terpinene‐_D_	Citrus and lemon aroma.
	Nonanal	Strong smell of grease and sweet orange.
	α‐Phellandrene	Black pepper and amiable lotus‐like aroma.
HJ4	Isobutanal	Sweet, dairy aroma with greasy notes of fat.
	Limonene	Turpentine with lemon flavor.
	Geraniol	Mild, sweet rose scent with a bitter taste.
HJ5	Linalool	With an aroma of lily of the valley.
	Isobutyl isobutyrate	Pineapple, grape skin aromas, and etheric notes.
	Ethyl formate Hexyl propanoate	Peach‐like odor, stimulating, astringent.
HJ6	Hexyl propanoate	Fresh scent with sweet fruits.
	α‐Pinene‐_M_	It has a piney, piney, and resinous odor.
	Isoamyl formate	There is a special plum and black spiky currant‐like sweet aroma.
HJ7	Propyl butanoate	Pineapple and almond like aroma, sweet banana, and pineapple fruit flavors.
	Myrcene‐_D_	It has a sweet orange flavor and balsamic notes.
	Ethyl acetate‐_D_	It has a fruity flavor.
HJ8	α‐Pinene‐_M_	It has a piney, piney, and resinous odor.
	Propanal	Strong musty odor from topical alcohol.
	Ethyl acetate‐_D_	It has a fruity flavor.
HJ9	α‐Pinene‐_M_	It has a piney, piney, and resinous odor.
	3‐Methyl‐2‐butanol	It has a fresh and fruity aroma.
	(z)‐3‐Hexenyl acetate	Intense aromas of freshly cut Liu grass and green leaves and fruits.
HJ10	Hexyl acetate	Delightful fruity aromas and pear‐like sweet and sour flavors.
	(Z)‐hept‐4‐enal	Grassy and oily aroma.
	Butanoic acid, 2‐methylpropyl ester	Apple and pineapple‐like aromas with rum‐like sweetness.
HJ11	Ethyl acetate‐_M_	It has a fruity flavor.
	Methyl acetate	Has a fragrant, fruity flavor.
	Propyl butanoate	Pineapple and almond like aroma, sweet banana, and pineapple fruit flavors.
HJ12	Myrcene‐_D_	It has a sweet orange flavor and balsamic notes.
	Nonan‐2‐one	Has a fruity aroma.
	Ethyl acetate‐_D_ Ethyl formate Ethyl formate	It has a fruity flavor.
HJ13	Ethyl formate	Peach‐like odor, stimulating, astringent.
	α‐Pinene‐_M_	It has a piney, piney, and resinous odor.
	β‐Ocimene	Grassy and floral with notes of orange blossom oil.
HJ14	Methional	Slightly sweet taste, with a special odor.
	2,3‐Pentanedione	Has a slightly sweet odor of quinone and a creamy odor in dilution.

HJ1–HJ14 were categorized into four groups (A: HJ1, HJ5, HJ10, H13; B: HJ2, HJ3, HJ4, HJ6; C: HJ7, HJ8, HJ9, HJ11, HJ12; D: HJ14) based on the PCA score plots of HJ1–HJ14. The classification trends of PCA for HJ1–HJ14 were described quadratically using OPLS‐DA analysis (Tu et al., 2022). OPLS‐DA, as a supervised discriminant analysis statistical method, can be used to model the correlation between VOC intensity and sample category. Compared to PLS‐DA discriminant analysis, OPLS‐DA analysis is more focused on modeling the data, making the classification trend more obvious (Boccard & Rutledge, 2013). The VIP method was used to explore the characteristic aroma substances, and the total sample size of the model was 28 (14 varieties × 2 replicates). The Y variables were the four types of huajiao, and the X variables were a total of 86 aroma substances that were used in the PLS‐DA model. The SIMCA14 plot is shown in Figure 9b. The OPLS‐DA score plot can intuitively reflect the differences between samples. The smaller the differences between samples, the closer the relative position in the plot, and vice versa (Ye et al., 2022). In the graph, principal component 1 accounted for 28%, and principal component 2 accounted for 20.8%. All four types of huajiao can be effectively separated in the graph, which indicates that the OPLS‐DA model of huajiao has achieved a better classification effect. In the OPLS‐DA model, R2 and Q2 are >50%, proving that the model's prediction is accurate (Bai et al., 2021). To test whether there is overfitting in the model, the OPLS‐DA model was subjected to a replacement test with parameter 200, and the test results are shown in Figure 9c. Q2 is −0.713, which proves that there is no overfitting in the model. The intensity of the effect of VOCs on group discrimination was evaluated by calculating the VIP value. The higher the VIP value, the greater the difference in aroma components between groups, and the more important it is to discriminate aroma types. It is generally believed that VIP > 1 indicates that this component can be used as a test substance (Xue et al., 2023). In the characteristic components of this paper, the VIP value >1.2 was used as the characteristic components for the classification basis of HJ1–HJ14. The VIP values of all volatile substances are shown in Figure 9d, and the VIP value of each substance is shown in the Appendix S1 (VIP table). In the VIP graph, α‐terpineol, geraniol, α‐phellandrene, butyl propanoate, linalyl acetate, 3,5,5‐trimethyl‐2‐cyclohexen‐1‐one, 3‐methylpentan‐2‐one, The VIP values of these components were greater than 1.2, indicating that these 7 VOCs can be used as key biomarkers to determine the distinction in the HPLS‐DA model of HS‐IMS. This constellation of VIP‐laden components predominantly comprises esters and terpenoids, reinforcing the capacity of these compounds to serve as geographical indicators. This inference harmoniously aligns with the preceding GC‐MS analyses. Identifying these significant biomarkers illuminates a pathway for discriminating the various “huajiao” types.

### 3.6 Correlation analysis between classification and geography of huajiao

**FIGURE 10:**
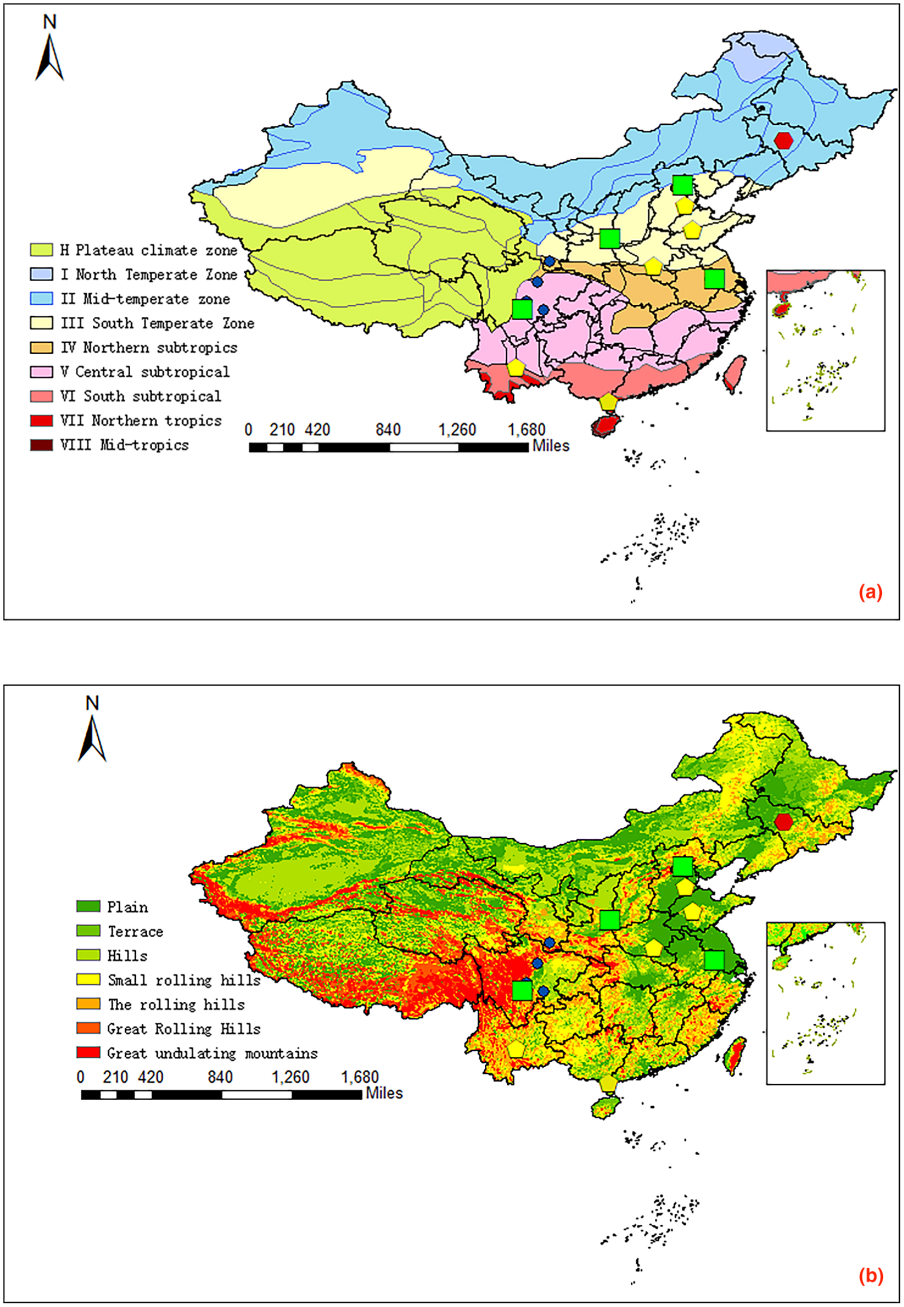
(a) Climatic map of HJ1–HJ14. (b) Topographic map of HJ1–HJ14.4. Conclusion.

As the Chinese saying goes, “Oranges are born in Huainan as tangerines, and tangerines are born in Huaibei as Hovenia.” This aphorism aptly underscores the profound impact of geographic distribution on species characteristics, a facet distinctly separate from germplasm considerations and shaped by the interplay of environmental and anthropogenic factors (Yang et al., 2022). The dispersion of species finds its roots in climate and soil conditions, the predominant environmental determinants of species distribution (Zhang et al., 2023). Figure 10 elegantly outlines the topography and climate intricacies underpinning HJ1–HJ14. In the figure, HJ2, HJ3, HJ4, and HJ6 are blue dots, and these species are located in the Sichuan Basin of China, with a warm climate and hilly terrain.HJ2, HJ3, and HJ4 are located in Sichuan Province, which has the same climatic elevation and many similar characteristics.HJ6 is in a different climatic zone, but all four huajiao species are in a subtropical monsoon climate in the general area. The geographic coordinates of HJ6 are similar to those of HJ2, HJ3, and HJ4, and the germplasm resources may be homologous, which is valuable for further research. HJ14 is labeled as a red hexagon and is located far from the coast in Jilin Province. It is similar to HJ1HJ5 in terms of topography. However, it is in the middle temperate zone and has differences with other huajiao in terms of precipitation, temperature, etc., which makes HJ14 have unique characteristics VOC different from HJ1–HJ13.HJ7, HJ8, HJ9, HJ11, and HJ12 are in one category and are labeled as purple pentagons in the figure. Among them, HJ8, HJ7, and HJ12 have similar geographic environments; the three are located in the plains, with mountains to the west and in the south temperate zone, which belongs to the monsoon climate zone.HJ8, HJ7, and HJ12 have a unique growth advantage in the summer due to the warm currents brought about by sea breezes from the Pacific Ocean. HJ11 is situated in the plains, close to the sea, and due to the high specific heat capacity of the ocean, which makes the temperature difference smaller, the rainfall is affected by the monsoon climate (Baldocchi et al., 2000).HJ9 is located in the rainforest area of Yunnan, which has a mild local climate (Yang, 2008).HJ9 and HJ11 have some similarities in precipitation and climate facets.HJ1, HJ5, and HJ10 are located at the edge of the precipitation curve in China, and all are blocked by high mountain ranges in the west. The summer monsoon winds are weakened, and rainfall increases after passing through these origins. The local heating effect of the mountain ranges causes a localized increase in temperature at the edge of the monsoon, reaching a similar environmental product (Boos & Kuang, 2010). The climatic environment of HJ13 is not identical to that of HJ1, HJ5, and HJ10, probably because of the superimposed effect of germplasm resources and geography, which makes HJ13 share the same taxonomic trend with HJ1, HJ5, and HJ10.

## 4 CONCLUSION

Huajiao not only adds aroma and flavor to food but also prolongs its shelf life and protects its safety. Therefore, huajiao is essential in the modern food industry and culture. To investigate the effects of different geographic environments in China on the aroma components of huajiao, this paper conducted an in‐depth study of 14 commercially available huajiao by using a variety of analytical techniques, including thermal analysis, GC‐MS, GS‐SPME‐IMS, and so on. The results showed that the content and proportion of VOCs of the huajiao varied.

Thermal analysis, a technique often used in materials science, is of great significance in exploring the thermal stability of VOCs. The different proportions of volatile substances in huajiao lead to differences in thermogravimetric data, which can be used to identify some huajiao. In addition, thermal analysis can directly observe the escape rate of aroma components of huajiao at different temperatures, which can be further explored in simulating the effects of other cooking methods on the aroma and sensory perception of huajiao.

Both GC‐MS and HS‐SPME‐IMS can effectively identify HJ1–HJ14. In this paper, the cluster analysis and correlation analysis of the Capsicum annuum data were carried out using CHIPLOT software. The analysis results showed that the classification results of GC‐MS and HS‐SPME‐IMS for huajiao were highly identical, indicating that both of them are highly sensitive in exploring the components of huajiao and can be used as an effective means for the separation and identification of huajiao. Finally, OPLS‐DA analysis was performed on the HS‐SPME‐IMS data of Capsicum annuum, and the model screened out seven key volatiles, including α‐terpineol, α‐phellandrene, 3‐methylpentan‐2‐one, geraniol, butyl propanoate, linalyl acetate, and 3,5,5‐trimethyl‐2‐cyclohexen‐1‐one.HJ1–HJ14 have a high degree of similarity in volatile composition but also have their unique aromas.

According to the analyses of HJ1–HJ14, the climatic environment and topographic conditions influence the classification of huajiao. The germplasm resources of huajiao have a decisive role in the content and type of VOCs. Still, among huajiao with similar germplasm resources, the VOCs show dependence on the climatic environment and topographic conditions. Among them, the climatic environment dominates and makes the GI more obvious. However, the sample size of huajiao in this paper is small, and the analysis of VOCs' dependence on climatic and topographical environments needs to be deeper, which leads to the inability to make a decisive judgment. In specific geographic settings, precipitation, average temperature, sunshine, and soil nutrients all impact the quality of huajiao. Therefore, further research could focus on verifying the effects of specific geographic environments on the odor characteristics of huajiao.

## AUTHOR CONTRIBUTIONS

**Dandan Chang:** Data curation (supporting); formal analysis (supporting); validation (supporting). **Yu Yang:** Methodology (supporting); visualization (supporting); writing – original draft (supporting). **Feiyan Tao:** Software (equal); supervision (equal); validation (equal). **Yu Ding:** Software (equal); supervision (equal); validation (equal). **Meiling Jian:** Investigation (supporting); supervision (equal); validation (equal). **Qinwan Huang:** Funding acquisition (supporting); project administration (supporting); writing – review and editing (supporting).

## CONFLICT OF INTEREST STATEMENT

The authors declare that the study was conducted without any potential conflict of interest arising from commercial or financial relationships.

## Supporting information


Appendix S1.

## Data Availability

The data that support the findings of this study are available on request from the corresponding author. The data are not publicly available due to privacy or ethical restrictions.
