# Supplementary material for: Correlation between climatic environment and characteristic components of 14 kinds of huajiao by thermal analysis techniques, GC‐MS and HS‐IMS
Source: Food Sci Nutr. 2024 May 3;12(7):4783–99. doi: 10.1002/fsn3.4126 (PMC11266924; doi:10.1002/fsn3.4126)
Supplement: Supplementary file 1 — Appendix S1. [file FSN3-12-4783-s001.zip › appendix file/Catalogue of annexes.docx]

Catalogue of annexes

In order to facilitate the reader's understanding of this paper's relevant experimental operations and data processing process, the group will be relevant to the design of this paper to all the data processing of the original documents and pictures are attached to the following table, I hope to be able to help you read.

| appendice | element | descriptions |
| --- | --- | --- |
| appendix file A | thermal analysis | Thermal analysis raw data for HJ1-HJ14 |
| appendix file B | GC-MS | GC-MS raw data and associated thermogram data for HJ1-HJ14 |
| appendix file C | HS-IMS | HS-IMS raw data and associated thermogram data for HJ1-HJ14 |
| appendix file D | PCA/OPLS-DA | Raw data and VIP analysis data from PCA and OPLS-DA analyses for HJ1-HJ14 |
| appendix file E | atlase | Topographic and terrain analysis maps for HJ1-HJ14 |
