# Supplementary figures and images for: Correlation between climatic environment and characteristic components of 14 kinds of huajiao by thermal analysis techniques, GC‐MS and HS‐IMS
Source: Food Sci Nutr. 2024 May 3;12(7):4783–99. doi: 10.1002/fsn3.4126 (PMC11266924; doi:10.1002/fsn3.4126)

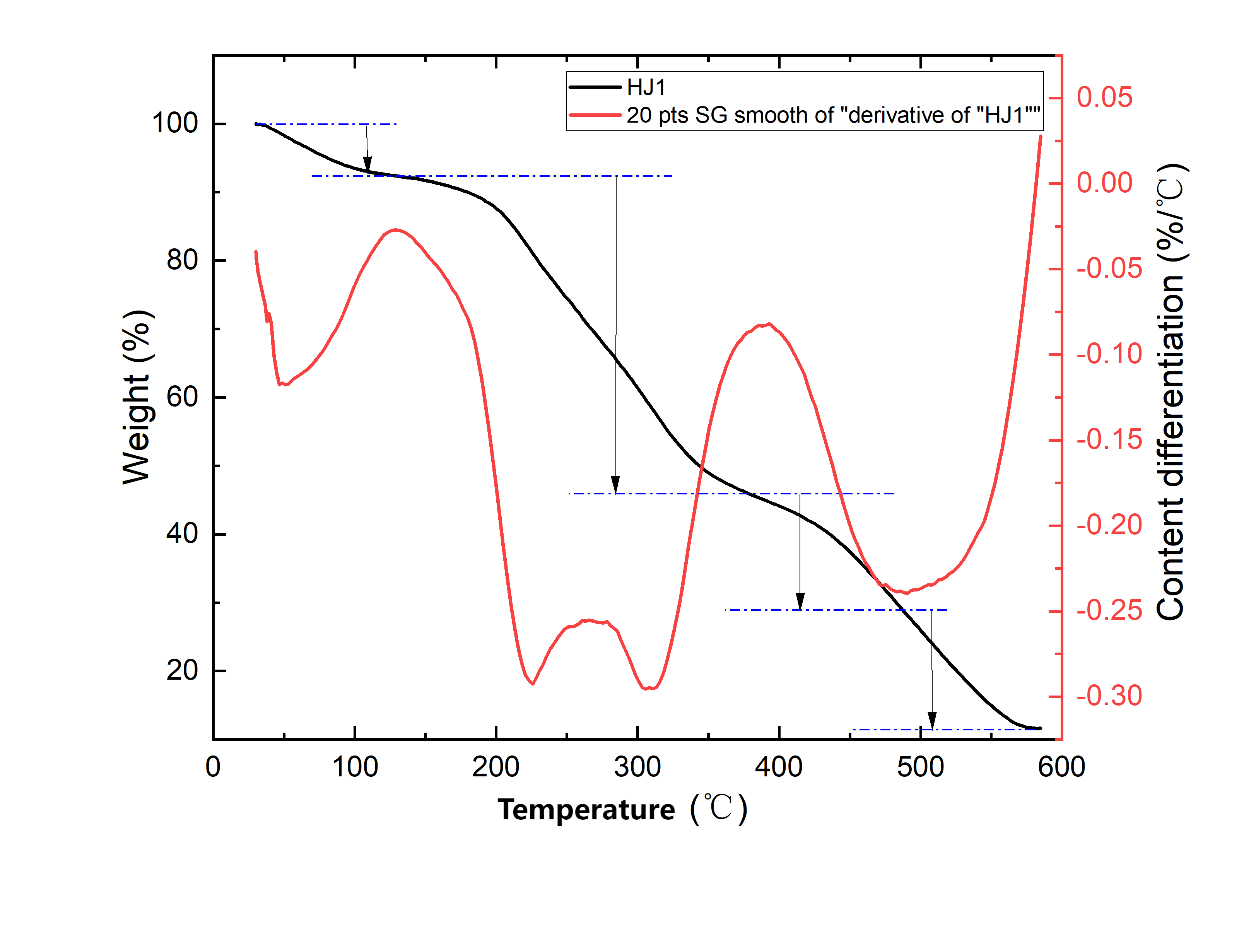

Supplement: Supplementary file 1 — Appendix S1. [file FSN3-12-4783-s001.zip › appendix file/appendix file A/figure/Fig.2(a).png]

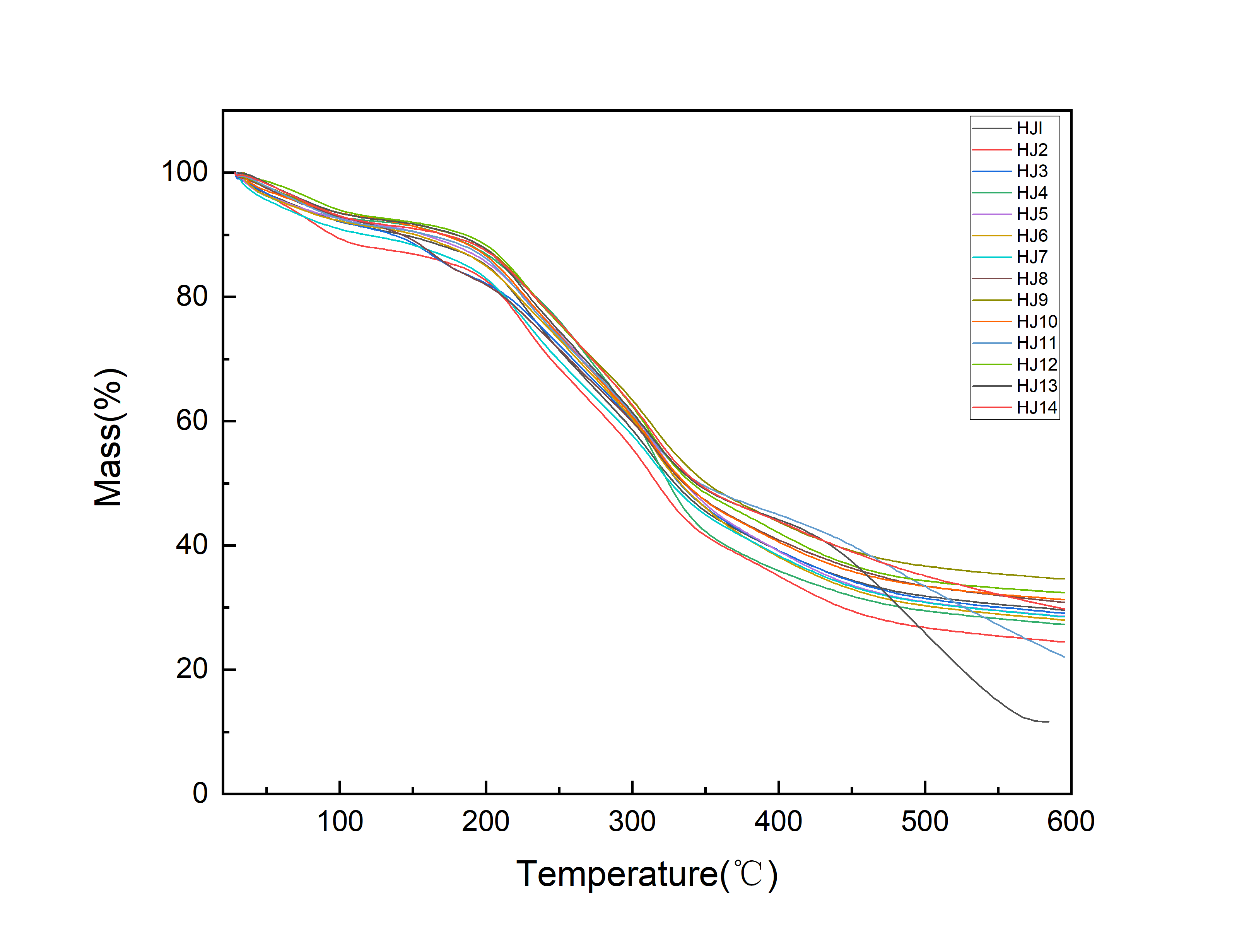

Supplement: Supplementary file 1 — Appendix S1. [file FSN3-12-4783-s001.zip › appendix file/appendix file A/figure/Fig.2(b).png]

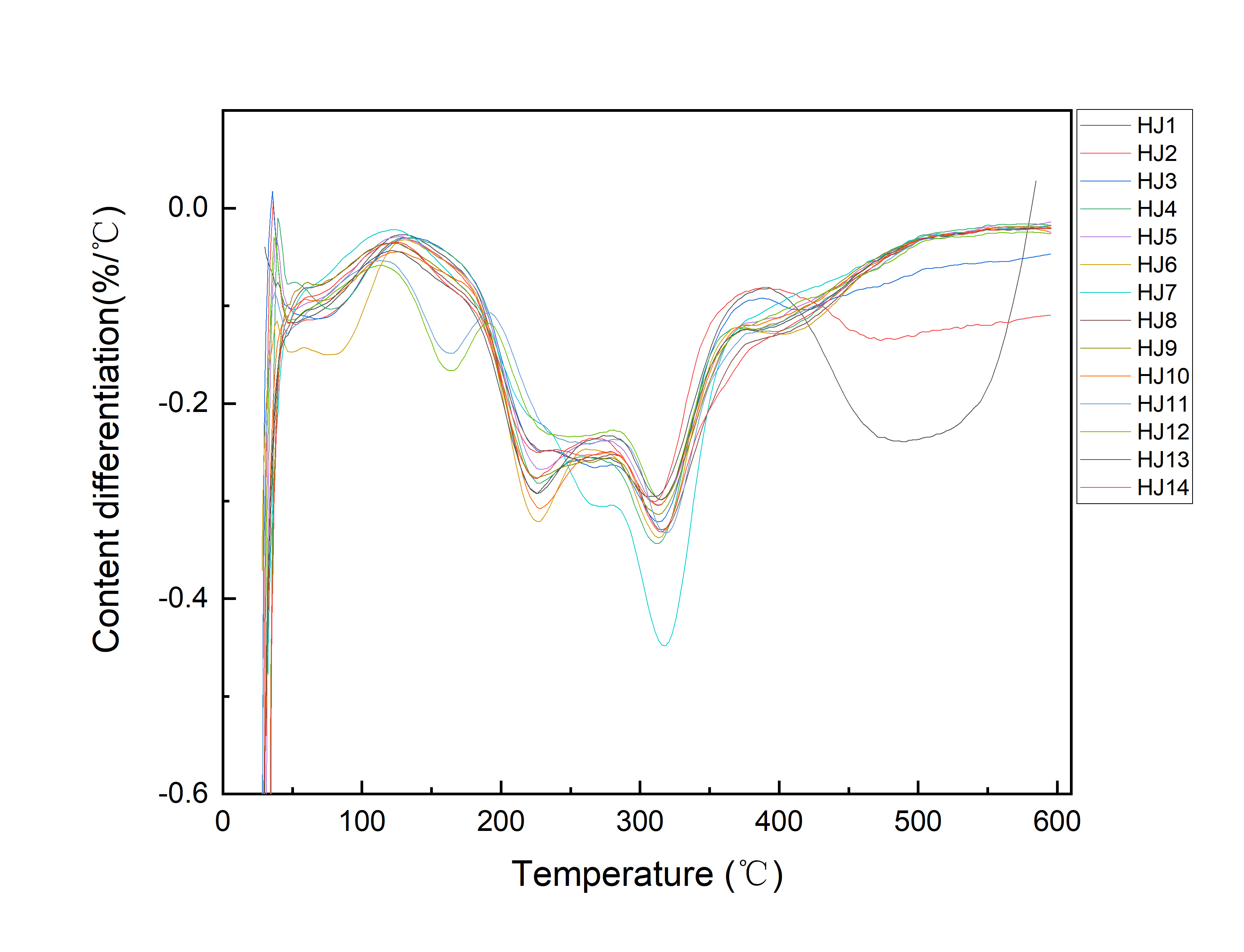

Supplement: Supplementary file 1 — Appendix S1. [file FSN3-12-4783-s001.zip › appendix file/appendix file A/figure/Fig.2(c).png]

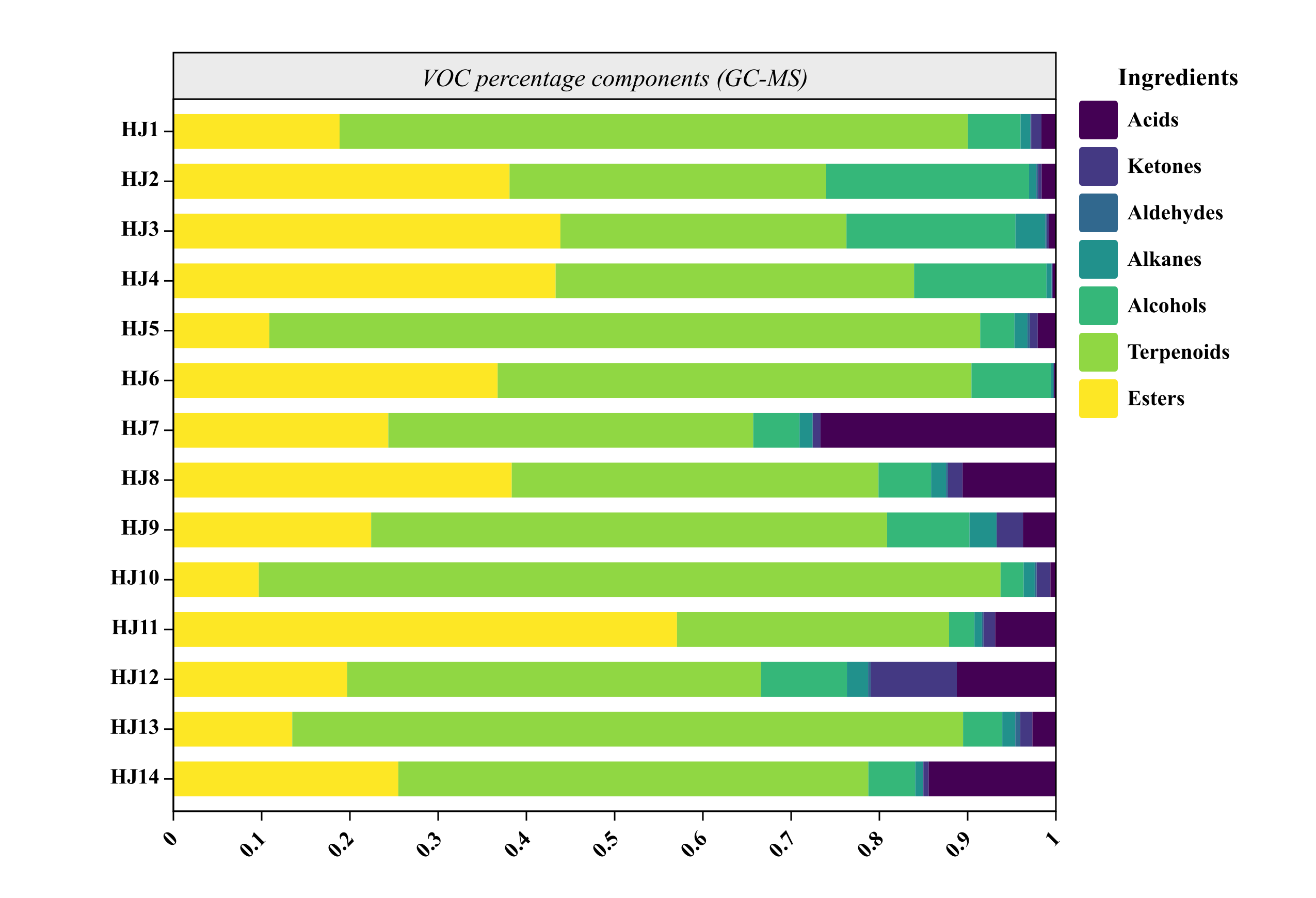

Supplement: Supplementary file 1 — Appendix S1. [file FSN3-12-4783-s001.zip › appendix file/appendix file B/figure/Fig.3.png]

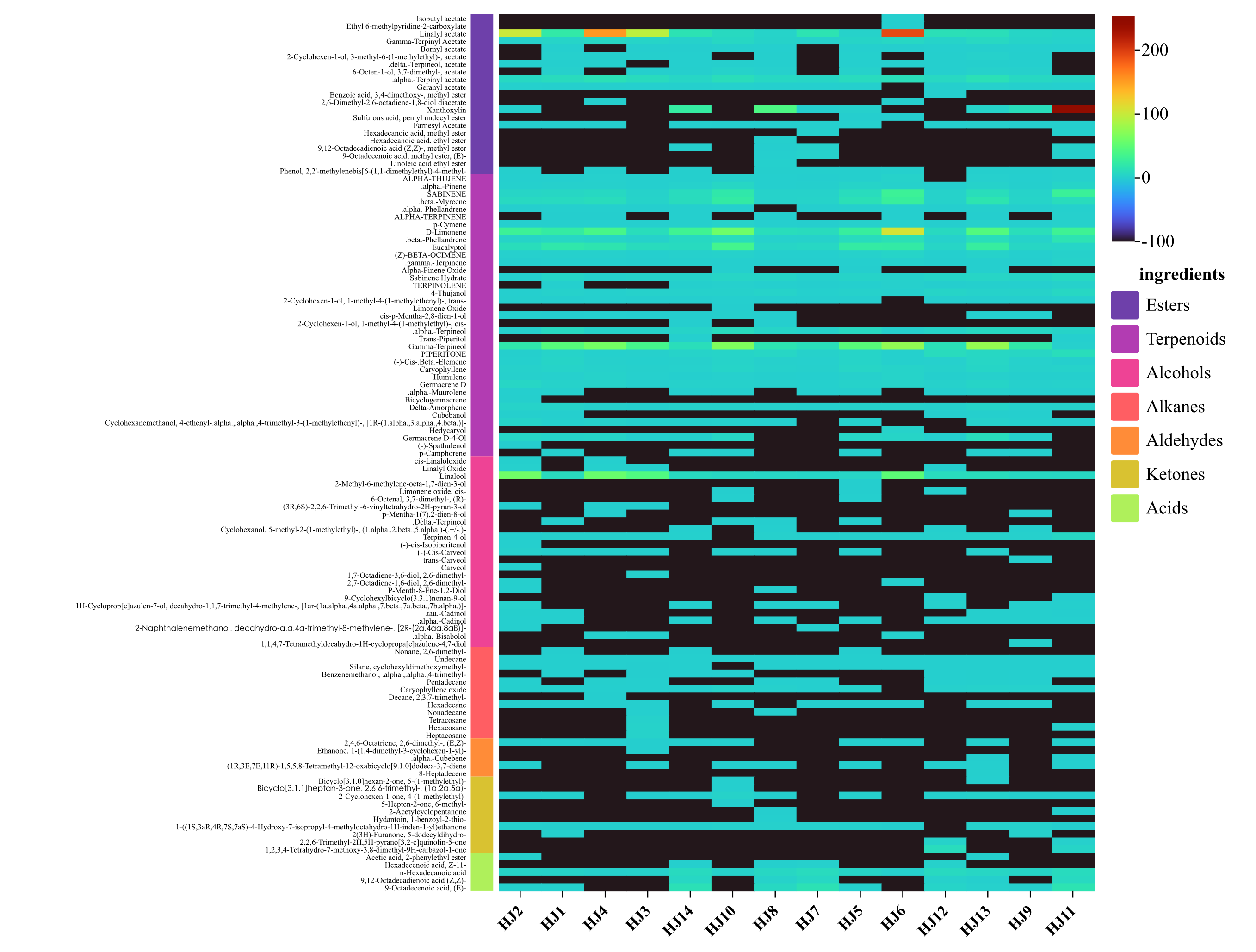

Supplement: Supplementary file 1 — Appendix S1. [file FSN3-12-4783-s001.zip › appendix file/appendix file B/figure/Fig.4.png]

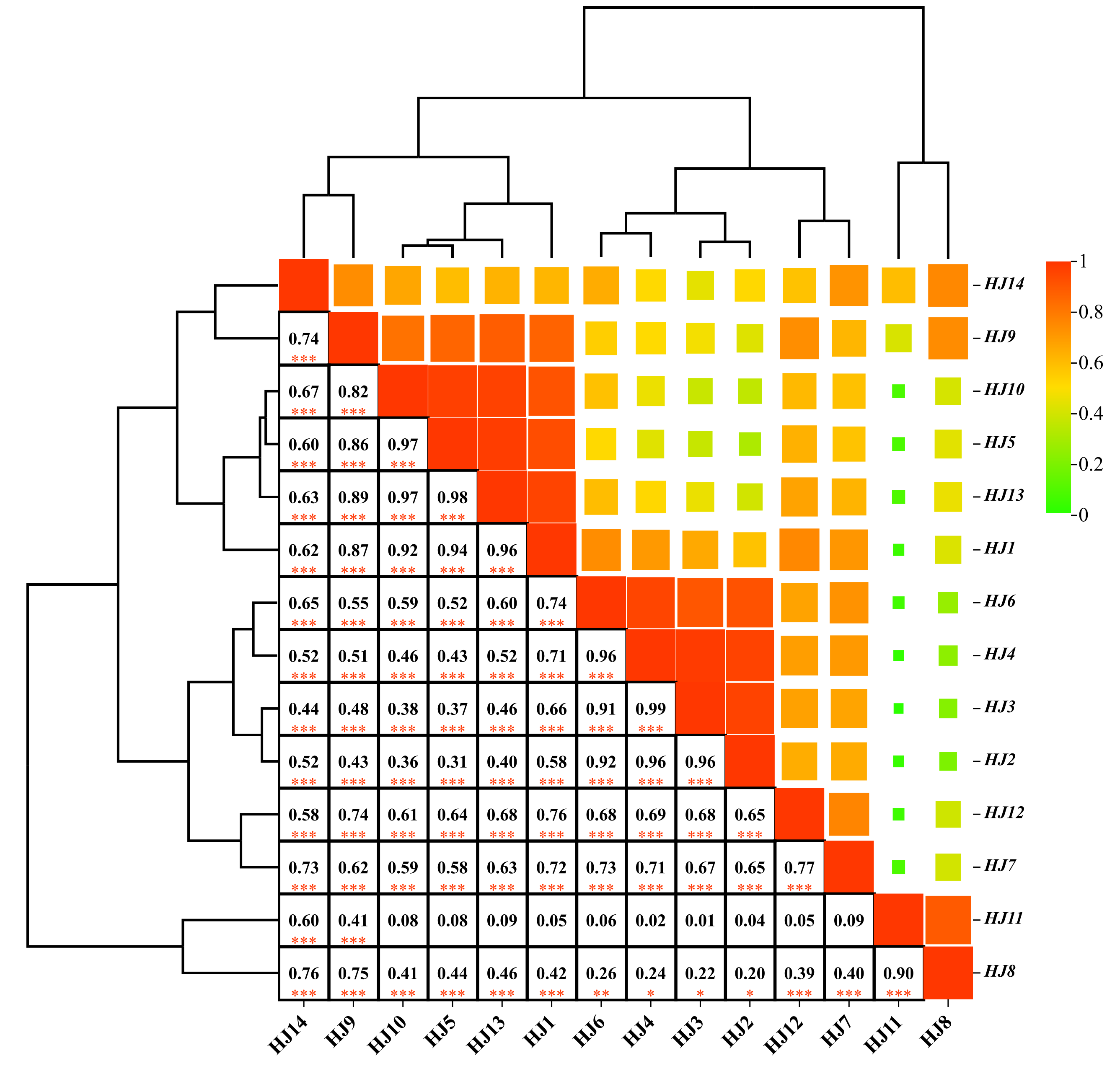

Supplement: Supplementary file 1 — Appendix S1. [file FSN3-12-4783-s001.zip › appendix file/appendix file B/figure/Fig.8.png]

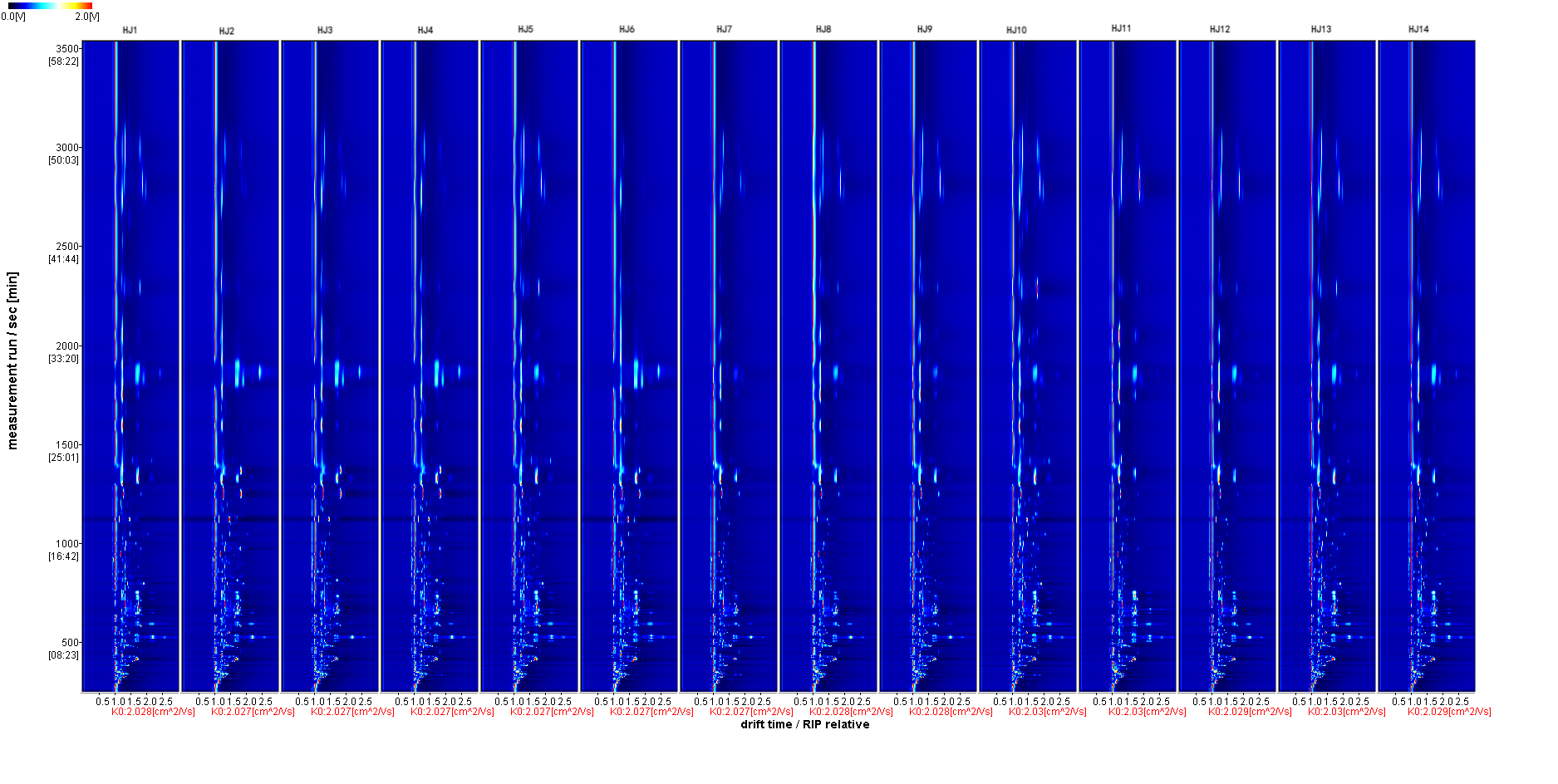

Supplement: Supplementary file 1 — Appendix S1. [file FSN3-12-4783-s001.zip › appendix file/appendix file C/figure/Fig.5(a).png]

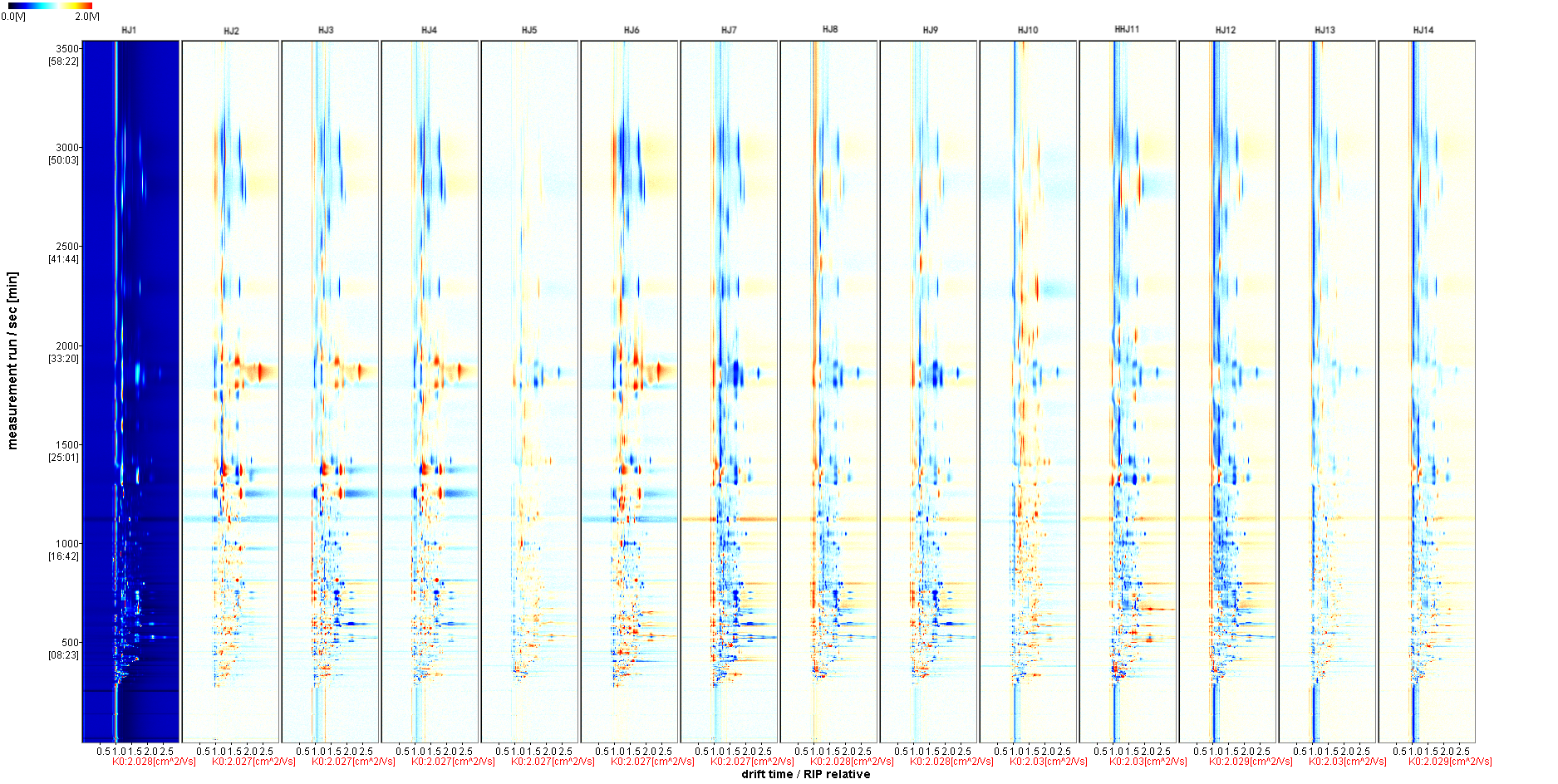

Supplement: Supplementary file 1 — Appendix S1. [file FSN3-12-4783-s001.zip › appendix file/appendix file C/figure/Fig.5(b).png]

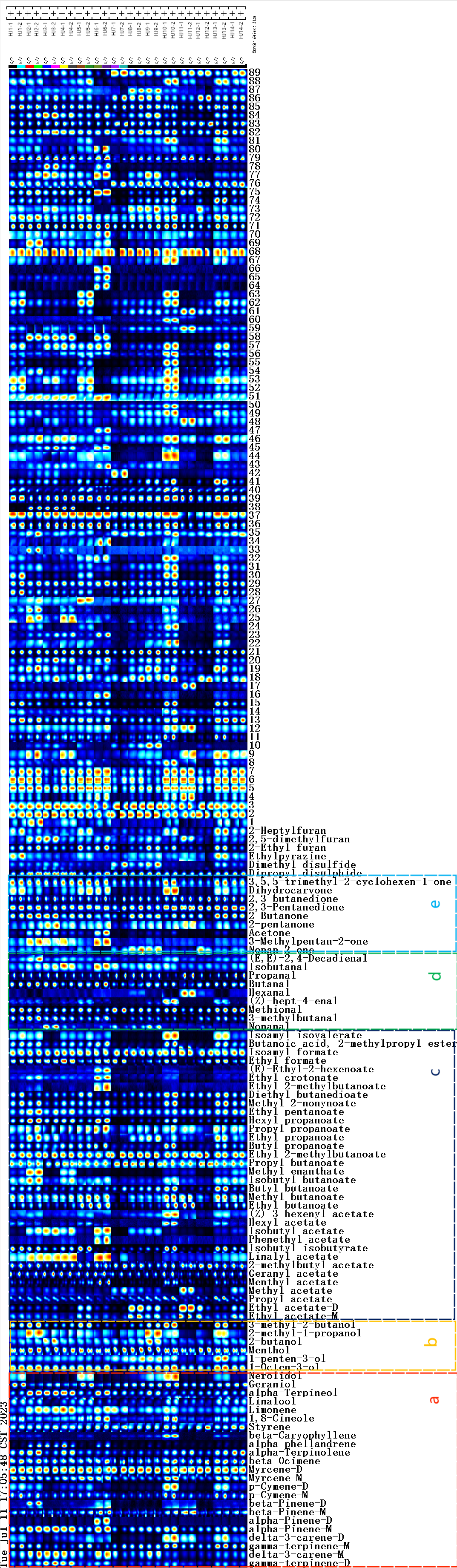

Supplement: Supplementary file 1 — Appendix S1. [file FSN3-12-4783-s001.zip › appendix file/appendix file C/figure/Fig.5(c).png]

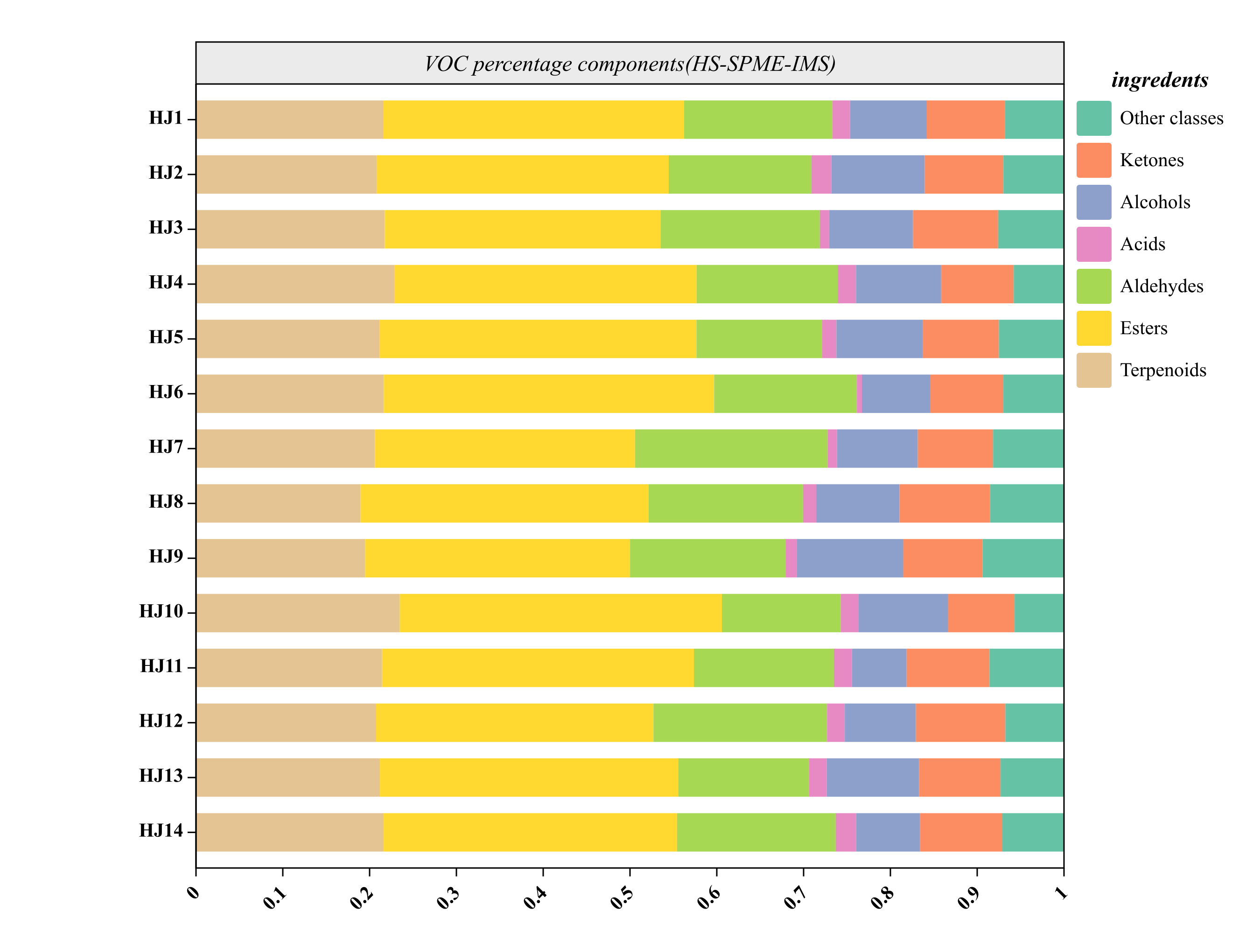

Supplement: Supplementary file 1 — Appendix S1. [file FSN3-12-4783-s001.zip › appendix file/appendix file C/figure/Fig.6 .png]

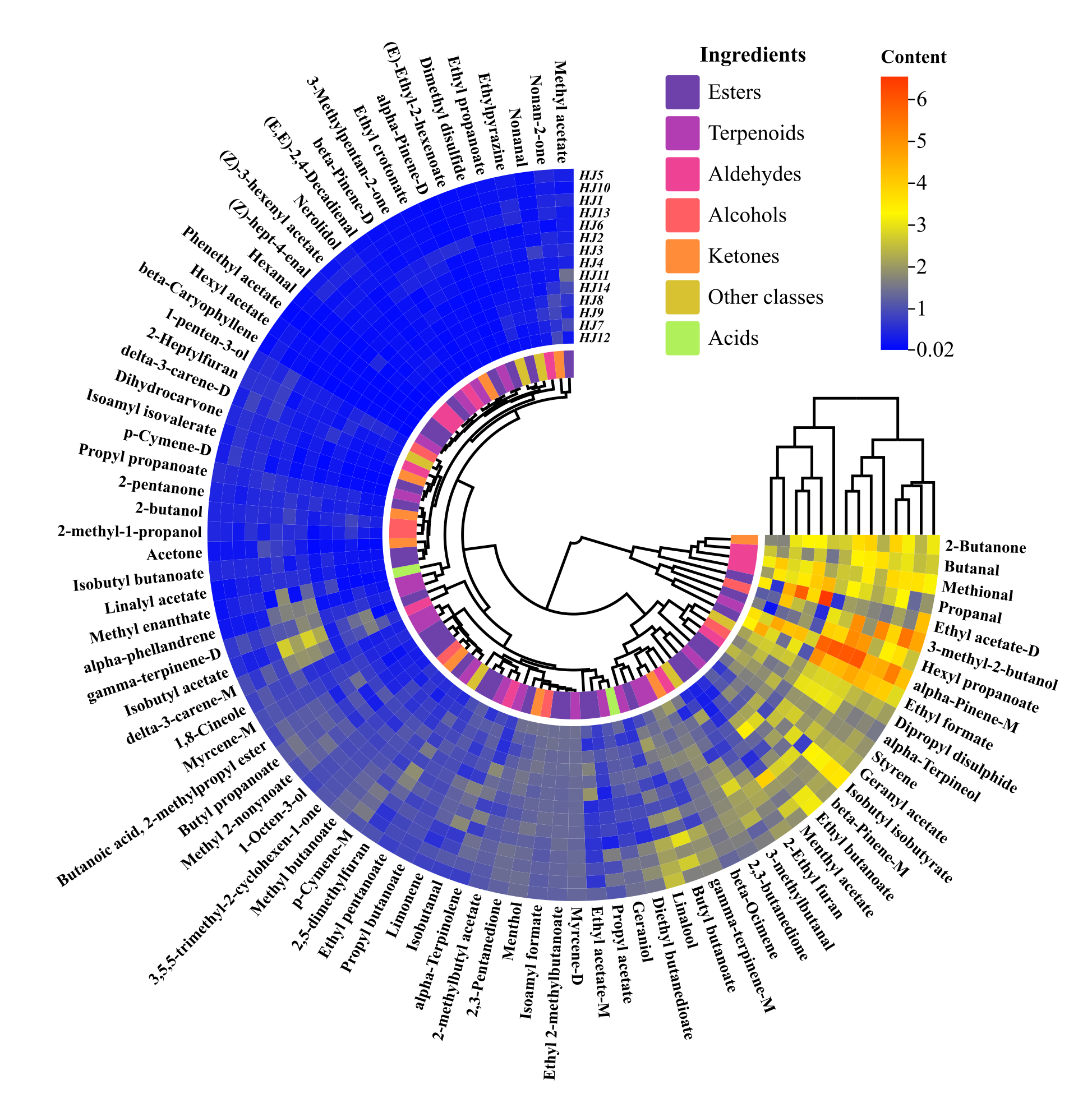

Supplement: Supplementary file 1 — Appendix S1. [file FSN3-12-4783-s001.zip › appendix file/appendix file C/figure/Fig.7.png]

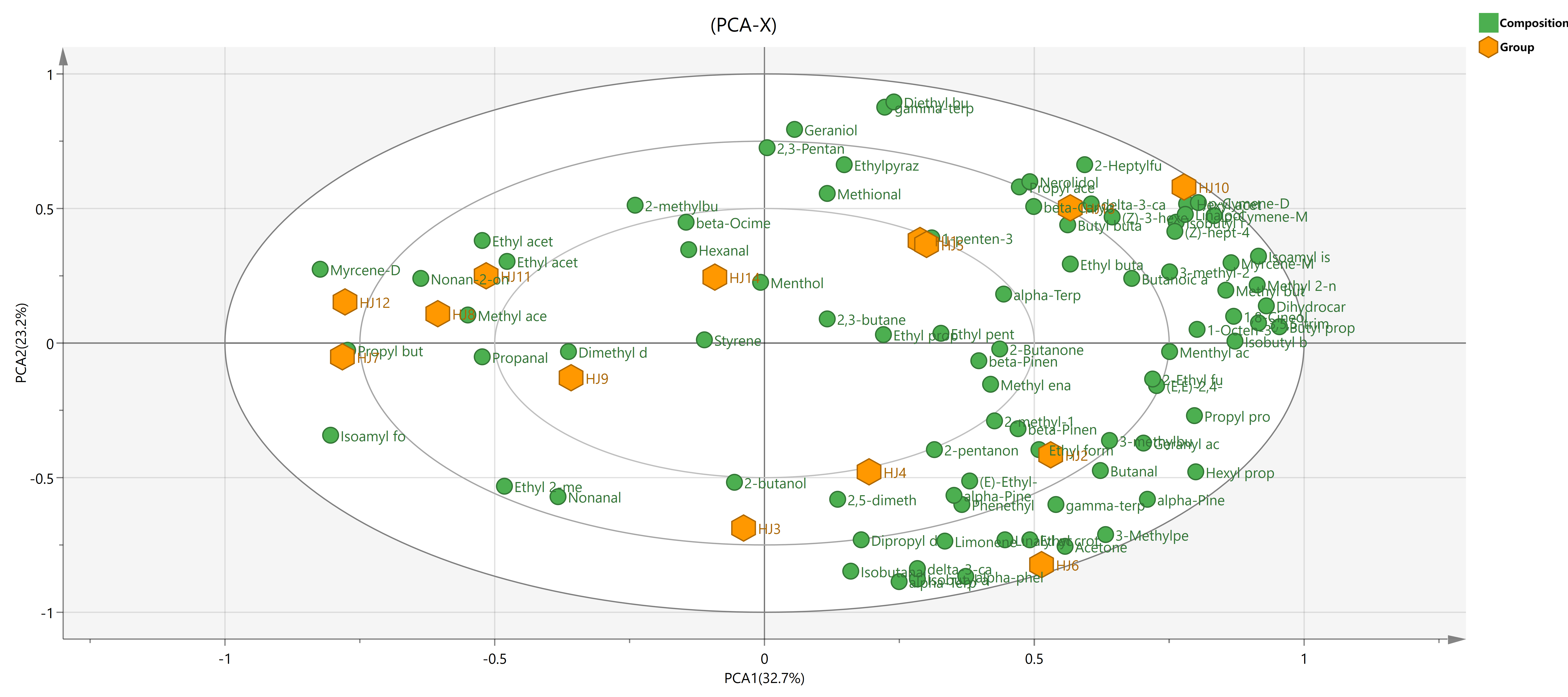

Supplement: Supplementary file 1 — Appendix S1. [file FSN3-12-4783-s001.zip › appendix file/appendix file D/figure/Fig.9(a).png]

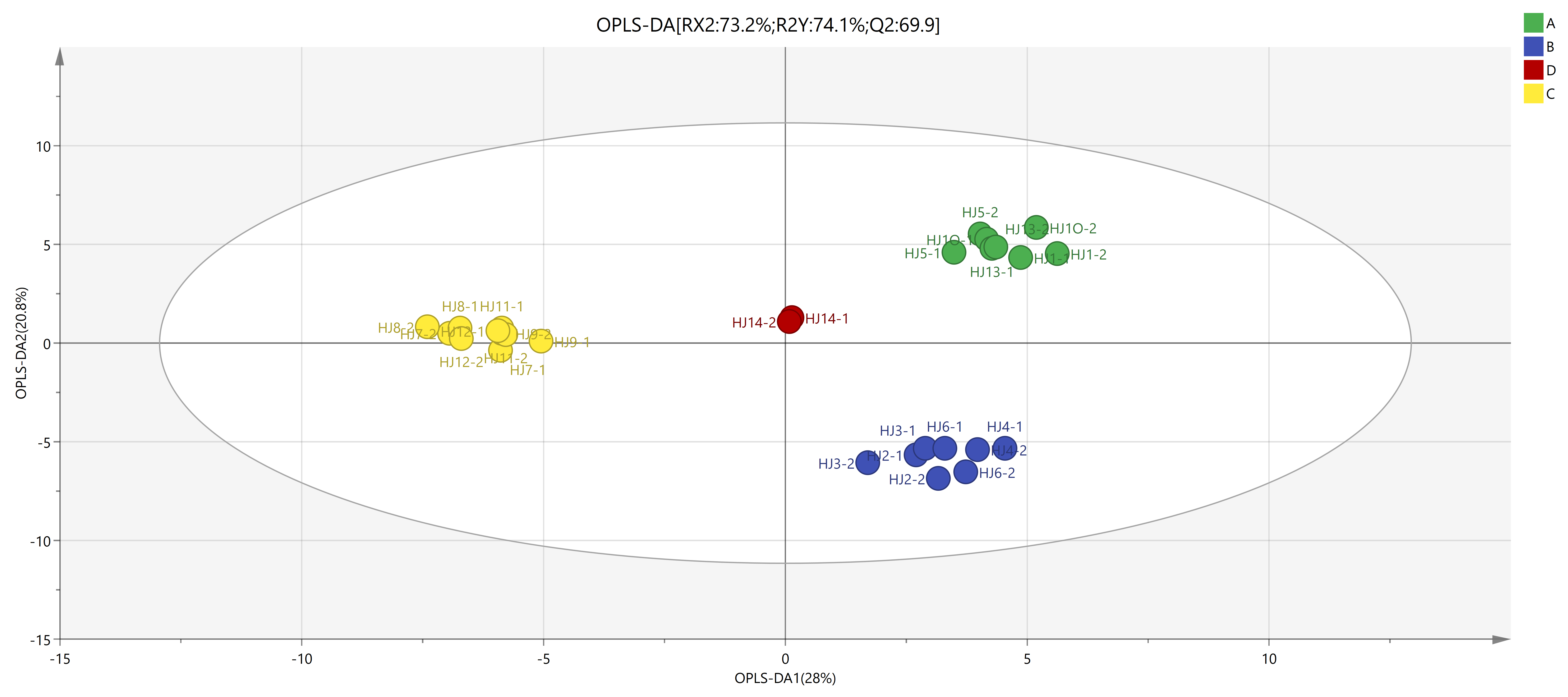

Supplement: Supplementary file 1 — Appendix S1. [file FSN3-12-4783-s001.zip › appendix file/appendix file D/figure/Fig.9(b).png]

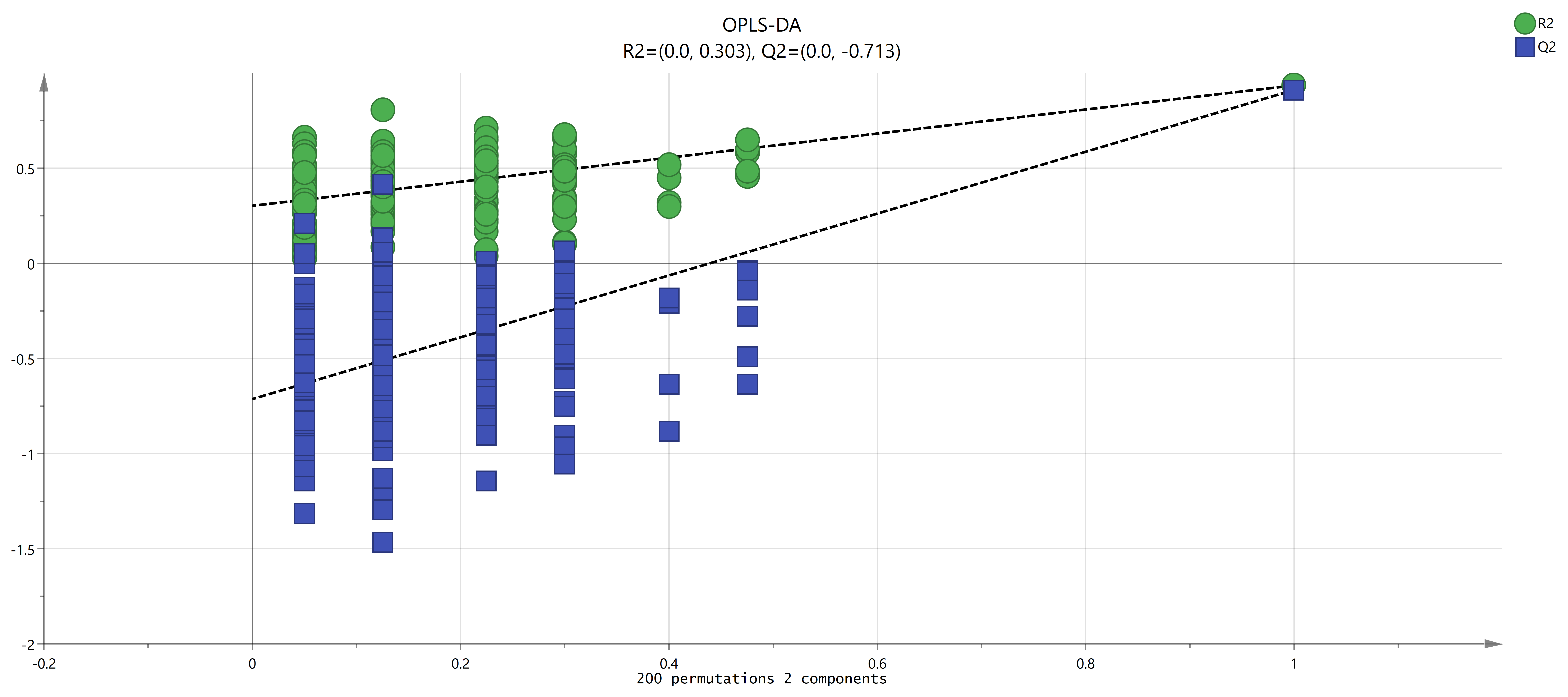

Supplement: Supplementary file 1 — Appendix S1. [file FSN3-12-4783-s001.zip › appendix file/appendix file D/figure/Fig.9(c).png]

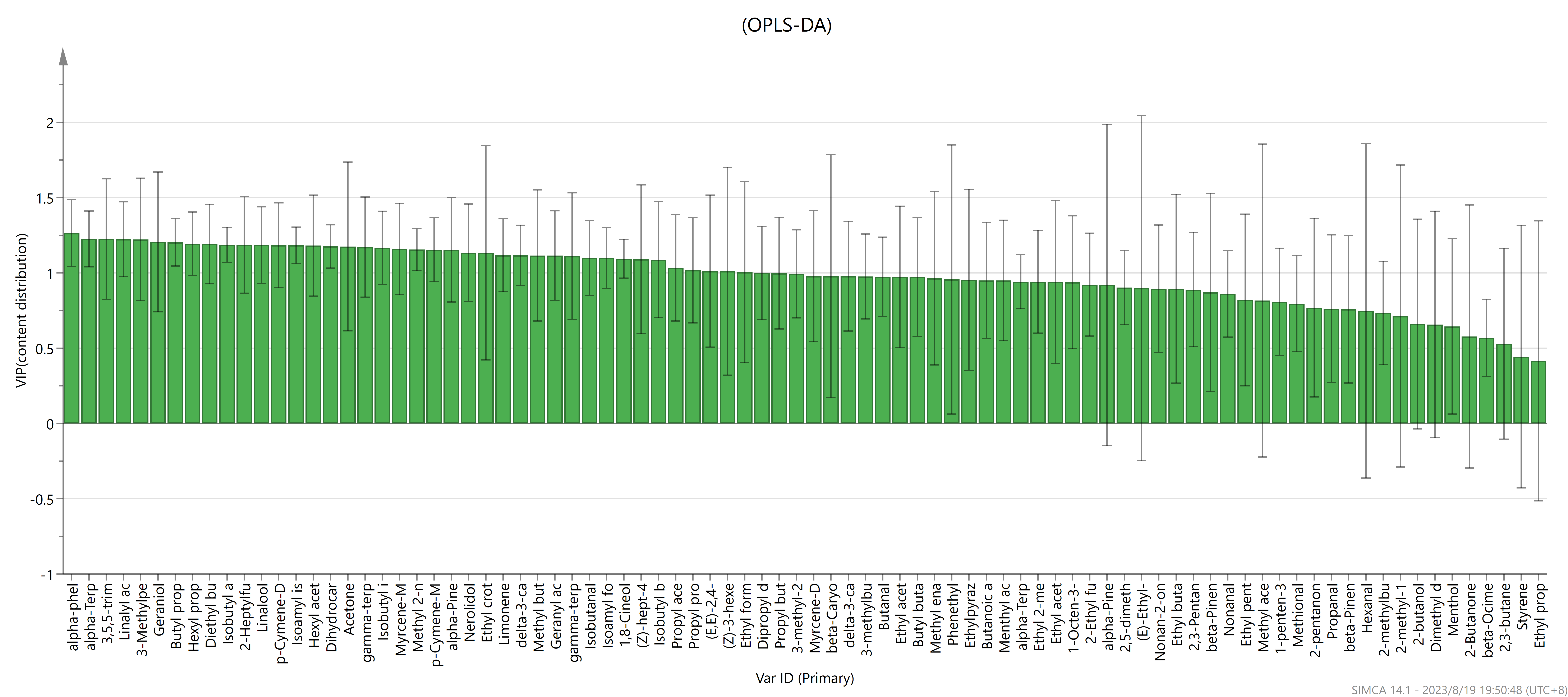

Supplement: Supplementary file 1 — Appendix S1. [file FSN3-12-4783-s001.zip › appendix file/appendix file D/figure/Fig.9(d).png]

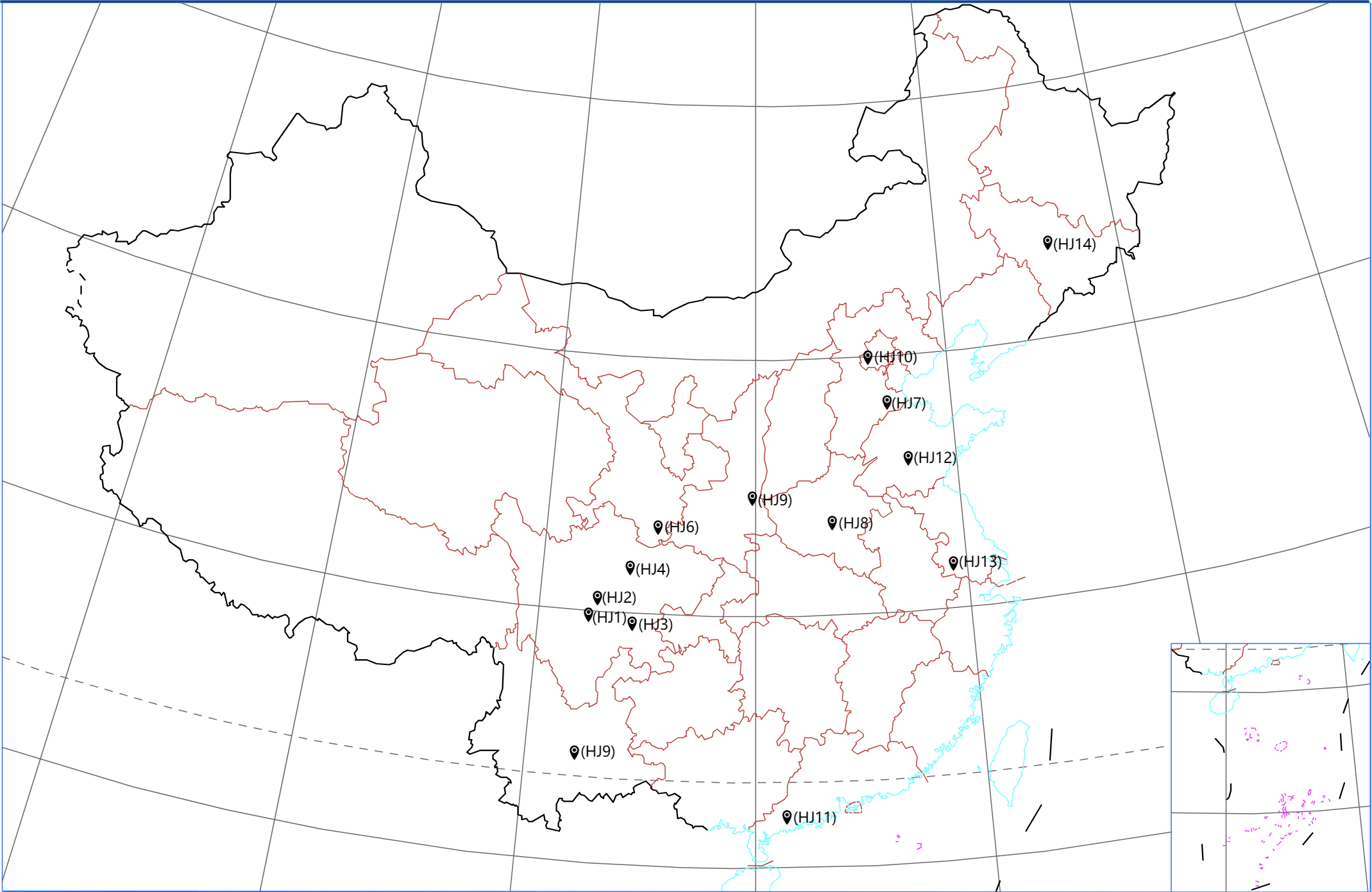

Supplement: Supplementary file 1 — Appendix S1. [file FSN3-12-4783-s001.zip › appendix file/appendix file E/figure/Fig.1.png]

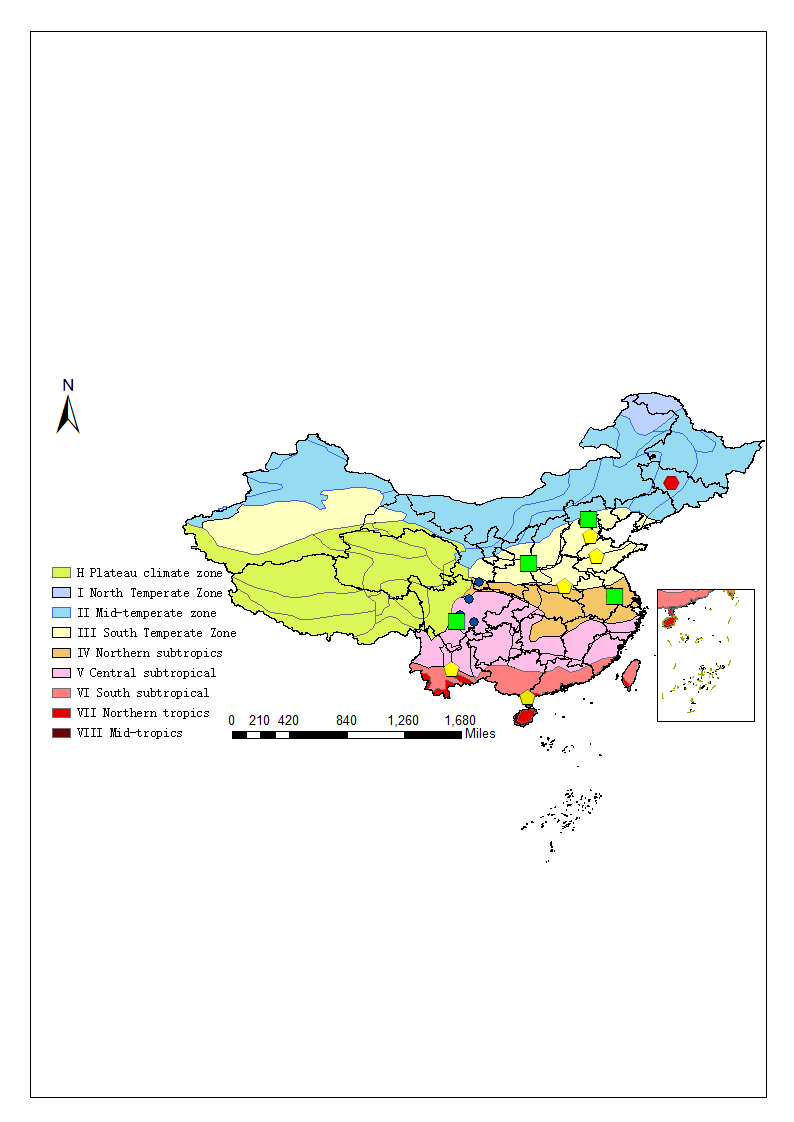

Supplement: Supplementary file 1 — Appendix S1. [file FSN3-12-4783-s001.zip › appendix file/appendix file E/figure/Fig.10(a).png]

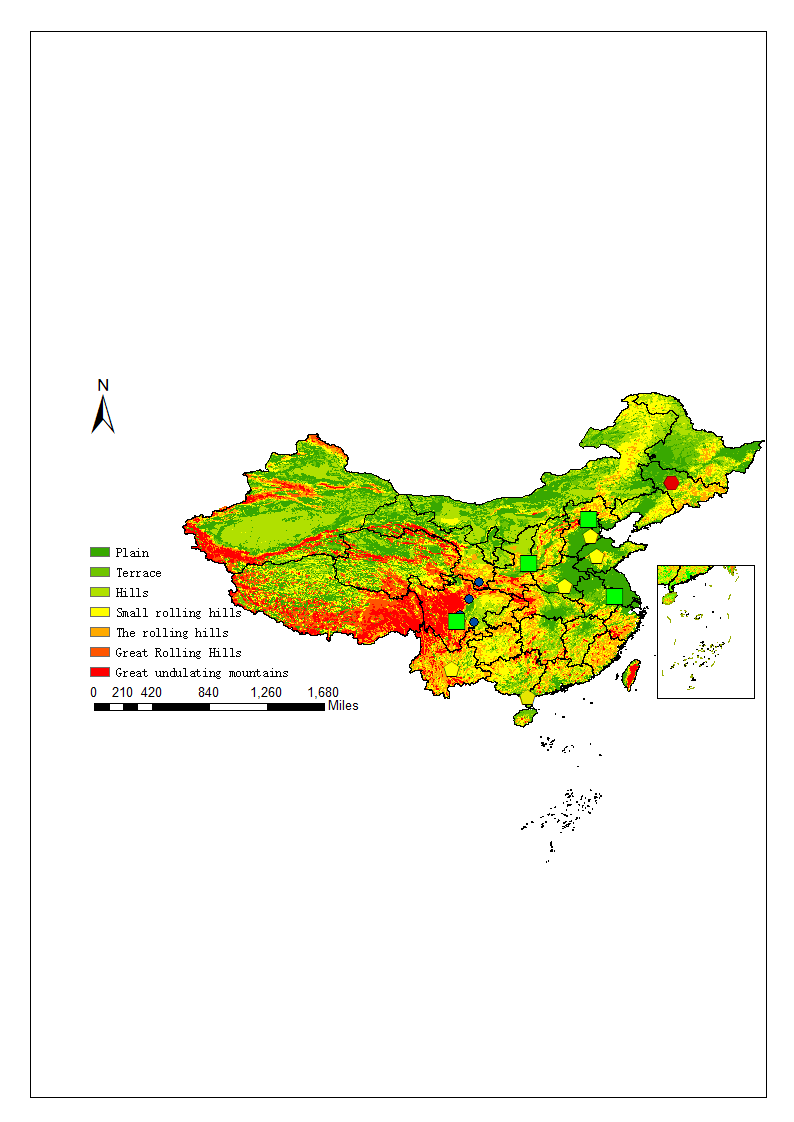

Supplement: Supplementary file 1 — Appendix S1. [file FSN3-12-4783-s001.zip › appendix file/appendix file E/figure/Fig.10(b).png]
